# Structure-guided identification of a potential inhibitor targeting the VacA toxin of *Helicobacter pylori*

**DOI:** 10.1371/journal.pone.0354383

**Published:** 2026-07-22

**Authors:** Farzana Akter, Md. Nur Islam, Md. Easin Mia, Md. Adnan Munim, Md. Murad Hossain

**Affiliations:** 1 Department of Biotechnology and Genetic Engineering, Noakhali Science and Technology University, Noakhali, Bangladesh; 2 Centre for Life-science Advancement in Bioinformatics (cLAB), Noakhali, Bangladesh; Kafrelsheikh University Faculty of Pharmacy, EGYPT

## Abstract

Various human diseases including gastric inflammation, ulcers, stomach malignancies, and even coronary heart disease are significantly influenced by the infection of the gram-negative bacterium *Helicobacter pylori.* While antibiotics are commonly used to treat *H. pylori* infection, their effectiveness is compromised by the development of antibiotic resistance. Therefore, the development of novel therapeutics against this pathogen is imperative. The virulence factor “vacuolating cytotoxin autotransporter (VacA)” was annotated from *H. pylori* genome by using the RAST server. All strains of *H. pylori* contain VacA toxin. Phyre2 and SWISS-MODEL servers were used for modeling and evaluating the structure of the active site containing p55 domain of VacA toxin (PDB: 2QV3) which shows the Ramachandran plot (98.68%), MolProbity Score (1.32), Bad Bonds (0), and Clash Score (5.78) of the refined model of VacA toxin. Autodock Vina was used to dock 3000 ligand molecules obtained from the ZINC15 database which identified only 178 ligands bind to VacA’s active site. Molecular docking and ADMET studies predicted ZINC4004291 ((3As,4R,9bS)-4-pyridin-4-yl-8-(trifluoromethyl)-3a,4,5,9b-tetrahydro-3H-cyclopenta[c]quinolone) as the best inhibitor of VacA toxin based on its strong binding affinity (−6.9 kcal/mol) and low toxicity. Molecular dynamics simulation indicated favourable levels of RMSD, RMSF, Rg, SASA and Hbonds exploring the stable dynamics of the docking complex between the p55 domain of VacA toxin and ZINC4004291 ligand. Moreover, principal component analysis and Gibbs free energy landscape studies indicate the favourable thermodynamic stability of the complex during 100 ns simulation period. Thus, the ligand ZINC4004291 is a computationally identified in silico hit for a possible drug to treat *H. pylori* infection, while additional experimental validation required.

## Introduction

*Helicobacter pylori* is a type of bacteria that is gram-negative, has a spiral shape, and prefers environments with lower levels of oxygen. It is typically found in the human stomach, where it resides*.* Initially, it was thought that the stomach’s unfavourable acidic environment prevented microbes from growing there. However, after the confirmation of the survival of *H. pylori* in the human gut was reported, several colonization techniques utilizing virulence factors were explored [1]. Highly diverse *H. pylori* bacteria exhibit different levels of virulence depending on where they are found [2,3]. In developed nations, the prevalence of *H. pylori* infection increases by approximately 1% with each additional year of age. Although the infection is uncommon among children, it has been shown that over half of the children acquire the infection by age 10, and more than 80% of individuals become infected by the age of 20 in developing countries. Geographic variation has been found to influence the variance in infection prevalence between countries, which has been linked to socioeconomic status, the degree of urbanization, and inadequate sanitation throughout childhood [2]. The variation in the prevalence of *H. pylori* infection among different countries worldwide was shown in a recent study where New Zealand had the lowest rate of 9.2% and a maximum of 87.8% infection was recorded in Northern Nigeria [4,5].

*H. pylori* plays a fundamental role in the growth of gastroduodenal reflux disease and is etiologically linked to stomach ulcers, primary gastric lymphoma with B-cells, and cancer of the gastrointestinal tract [2]. *H. pylori* infection has been found to coexist with numerous other medical conditions, including gastritis, ulcerative colitis, gastric carcinoma, non-ulcer indigestion, gastrointestinal ulcer, stomach lymphoma originating from mucosa-associated lymphoid tissue, as well as cardiovascular disease [6]. Infection with *H. pylori* is related to more than 90 percent of stomach ulcers and over 95 percent of duodenal ulcers, and their rate of recurrence significantly decreased after the course of *H. pylori* elimination [6]. Therefore, *H. pylori* has been classified as a class I carcinogen by the World Health Organization (WHO) due to its well-established role as the primary cause of stomach cancer [7].

Even though the host immune system typically eradicates pathogens, *H. pylori* has evolved several defence strategies that evade both innate and adaptive immunity, allowing it to persist within the host. This bacterium can change the compounds on its surface to avoid being recognized by innate immune receptors and alters effector T cell function to thwart the adaptive immune responses [8]. The formation of various virulence factors is one of the key tactics adopted by *H. pylori*. The progress of gastric disease is influenced by the presence of bacterial colonization components (like BabA, SabA, OipA, and HopQ), effector molecules (like CagA, VacA, and HtrA), and outer membrane vesicles [2]. The bacterial virulence factor cytotoxin-associated vacuolating autotransporter plays a pivotal role in *H. pylori*-induced pathogenesis [9,10]. A 140 kDa protoxin called vacuolating cytotoxin autotransporter (VacA) is released into the intestinal lumen by *H. pylori* [10,11]. During the secretion process, it undergoes p33 domain and p55 domain cleavage, producing a mature 88 kDa toxin. The p55 region contains one or multiple domains responsible for binding to cells, while the N-terminal p33 domain is associated with the creation of pores [10,11]. VacA is classified as an atypical or non-classical pore-forming toxin due to its capacity to establish hexameric anion-selective channels within biological membranes. The name of the toxin is derived from its ability to induce the formation of “vacuoles” in the cytoplasm of gastric epithelial cells [1]. Along with its primary function, vacuolation, VacA has a number of other impacts on its target cells. In addition to altering the penetrability of the mitochondrial membrane, depolarizing the cell membrane, and causing the cell to separate from the basement membrane, VacA can also activate mitogen-triggered protein kinases, prevent antigen presentation, prevent T cells from activating and proliferating, and cause cell death [11]. Consequently, pathogenic effects such as gastritis, peptic ulcer syndromes, and several varied outcomes connected to adenocarcinoma in the human gastrointestinal tract occur.

With the advancement of medical research, there are a number of antibiotic-based treatments available to prevent the ailments created by *H. pylori* infection. Amoxicillin, clarithromycin, metronidazole, tetracycline, and bismuth are the antimicrobials that are most commonly utilized in the treatment of *H. pylori* related complications [12,13]. However, these treatments encounter intrinsic challenges, such as antibiotic resistance and its associated side effects, the potential for reinfection, and the considerable expenses associated with antibiotic-based therapy. To enhance the efficiency of drug research and development, computational algorithms, along with 3D visualizations and advanced software tools, have been integrated to support informed decision-making in designing or modifying molecules with targeted biological activity. Computer-Aided Drug Design (CADD) involves the application of computational tools and technologies to assist in the design and optimization of pharmaceutical compounds. Molecular docking and molecular dynamics (MD) simulation techniques are integral components of CADD, which involves the investigation of the compatibility and interaction between two or more molecular structures, such as a drug and an enzyme or protein. Current drug discovery can be delineated into six consecutive phases, which encompass target selection, target validation, recognition of lead compounds (ligands), refinement of lead compounds, preclinical evaluations, and clinical trials [14]. With the help of computational tools, it is possible to study, evaluate, and interpret the collaboration of medications (or potential drugs) with the target molecules in great detail, which is the main focus of contemporary methods for drug design and development [15].

Enormous development has been achieved in determining the pathogenesis of *H. pylori* infection. Currently, many antimicrobial therapies are available, but there is still no ideal treatment, and no indications for effective therapy continue to evolve [16]. Although substantial progress has been made in understanding the structural biology of VacA, including the crystal structure of the VacA p55 domain and cryo-EM models describing its oligomeric assemblies, however, most studies have primarily focused on elucidating toxin structure and mechanism rather than identifying small-molecule inhibitors targeting this virulence factor [17–19]. Moreover, structural analyses have further demonstrated that VacA assembles into oligomeric complexes that form membrane channels responsible for cellular vacuolation and cytotoxic effects. Despite these advances, there remains a translational gap between structural characterization of VacA and the development of targeted inhibitors capable of disrupting its function. In this context, the present study aims to address this gap by applying a computational drug discovery framework to identify potential small-molecule binders targeting the VacA p55 domain, thereby providing hypothesis-generating leads for future experimental validation. Hence, this study was design to identify potential small-molecule inhibitors predicted to interact with the VacA p55 domain through computational analyses.

This approach offers distinct advantages over traditional antibiotic treatments, such as decreased selection pressure for antibiotic-resistant varieties and limited disturbance to the host organism’s microbiota. Through the inhibition of virulence factors, such as extracytoplasmic molecules, these therapies aim to disrupt the bacteria’s ability to induce disease without necessarily eradicating it entirely. By integrating bioinformatics tools along with molecular docking and dynamics simulation procedures, researchers can accelerate the drug discovery process, potentially resulting in more potent treatments with fewer adverse effects. This work identified a drug candidate that may interfere with VacA-mediated host interactions and the survival of *H. pylori*, although the experimental validation remains pending. Additionally, this research stresses the significance of pinpointing specific drug targets within *H. pylori* proteins encoded by the core genome, which are not present in the corresponding host and resident microbiota, to devise highly selective therapies for *H. pylori* while minimizing off-target effects. Overall, this current study presents a comprehensive and potentially useful approach to tackling the challenges of *H. pylori* eradication, offering promising strategies for the development of alternative therapies with an extended window of research.

Although this study aimed to identify potential novel drug candidates against *H. pylori*-mediated infection by employing a combination of molecular docking and molecular dynamics simulation approaches, it presents a computational lead discovery framework targeting the VacA p55 domain. The findings are predictive in nature and require biochemical, cellular, and *in-vivo* validation before clinical application.

## Materials and methods

### Genome sequence analysis

**Fig 1:**
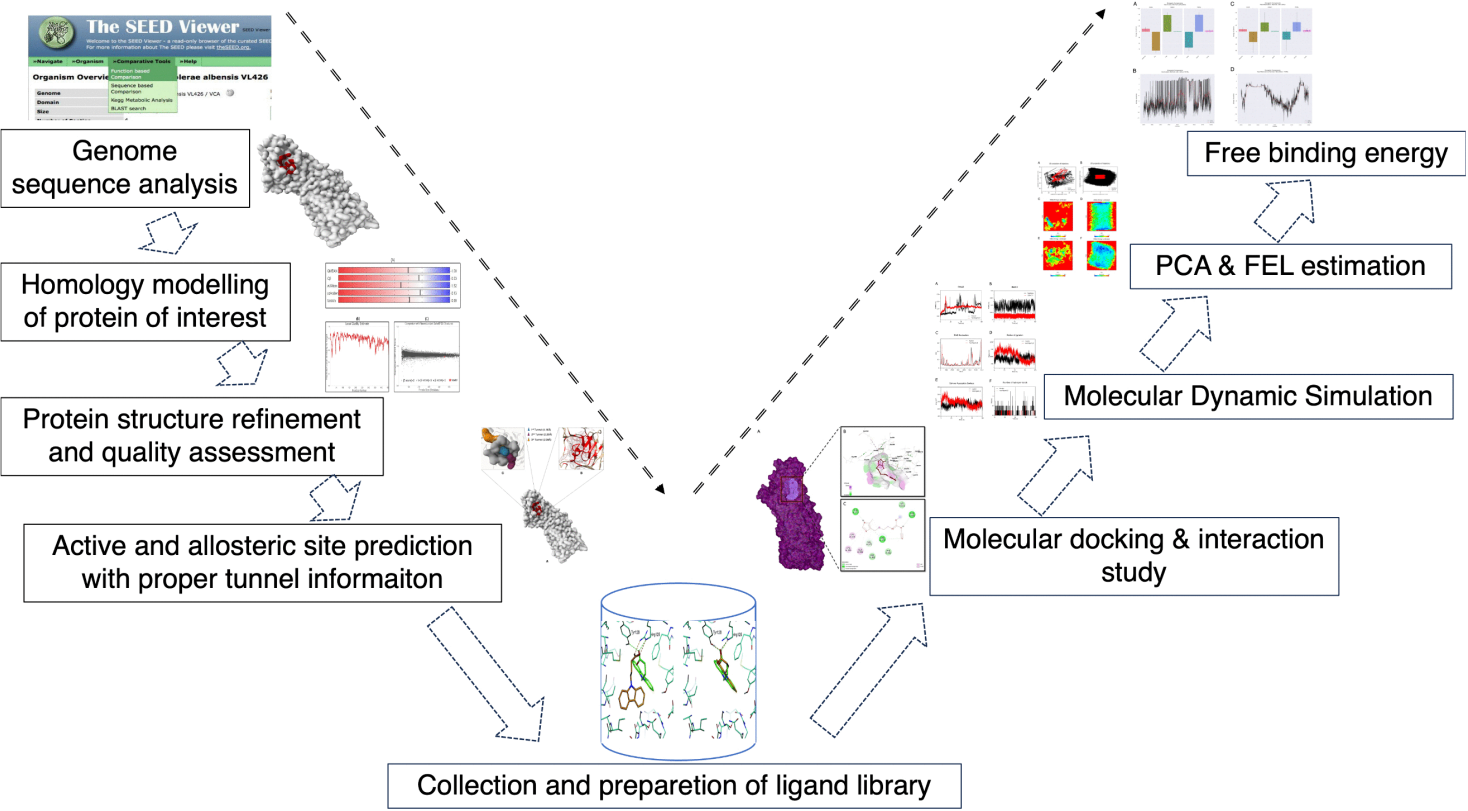
Flow chart of the study plan that conceptualizing the whole experiment.

The FASTA sequences of all the strains of the *H. pylori* genome were downloaded from the NCBI (National Center for Biotechnology Information) database. After downloading the genome sequences, the annotation process proceeded by uploading it to the Rapid Annotation utilizing the Subsystem Technology (RAST) server. The virulence characteristics of the bacteria were evaluated using RASTtk and SEED viewer [20,21]. A detailed flow chart of the study is shown in Fig 1.

### Protein preparation and active site prediction

The VacA toxin protein structure was retrieved from the RCSB Protein Data Bank (PDB), and the p55 domain was specifically selected for analysis. The crystal structure of the p55 domain (S1 Fig) of the *H. pylori* VacA toxin is designated by the PDB identification number (PDB ID) 2QV3. The homology modeling of VacA was carried out by the Phyre2 (S2 Fig) protein folding recognition server with the sequence retrieved from the PDB server [22]. Following homology modeling, the VacA structure was refined using the GalaxyRefine server (S3 Fig). The ‘GalaxyWEB’ server utilizes template-based modeling to predict protein structure from its sequence and also enhances loop or terminus regions through ‘ab initio modeling’ techniques. [23,24]. SWISS-MODEL server was then used for the structural assessment of the refined protein [25,26]. The Ramachandran plot score, MolProbity score, Quality estimation, and Residue quality of the protein were used to assess the structure [27]. The refined structure was then prepared with Chimera for charge field application and the addition of hydrogen atoms. Before docking, the target protein’s active site and the responsible amino acid positions were identified using the CASTp server and P2Rank suite of the PrankWeb tool [28,29].

### Ligand preparation

Potential ligands were downloaded from the ZINC15 database server. ZINC’s free database offers over 230 million commercially available compounds in ready-to-dock 3D formats for virtual screening purposes [30]. The downloaded files from the ZINC server were transformed into a ligand library in sdf format by using Microsoft Powershell. Open Babel was used to minimize ligand energy before converting molecules to PDBQT format in PyRx. In addition, fragments smaller than a specified size were removed using OpenBabel’s StripSalts function, and partial charges were assigned using OpenBabel or PyBabel from MGLTools as part of the preparation process [31]. “Lipinski Rule of Five” was applied for all the ligands to distinguish between molecules that are similar to drugs and those that are not [32,33].

### Control molecule selection

A reference compound that serves as a standard for assessing the binding affinity and interaction profile of proposed ligand molecules in *in silico* studies was chosen based on its molecular weight and structural stability with the target protein. It offers a comparative framework to evaluate the relative performance of our proposed ligand in terms of docking scores, binding affinity, and key molecular interactions. The selection was usually guided by existing drug databases available in the market to treat different gastric conditions, ensuring the control molecule has validated experimental or clinical relevance to the target of interest. Though ranitidine is not directly used as a drug for combating *H. pylori*, however, this choice was made only to provide a profile of comparative computational benchmark along with strong pharmacological relevance.

### Molecular docking

Docking is a technique used to predict the most favourable orientation of a ligand when bound to a receptor, leading to the formation of a stable complex. Scoring functions are utilized to predict the binding affinity between two molecules. The preparation of the docking process started with protein and ligand energy minimization. Dockman- PyRx software (version 1.2) was used for rapid molecular docking. A large molecular screening was used for docking 3000 prospective ligand molecules obtained from ZINC15 database. This tool was designed to apply an automated grid map calculations and arranges the outcomes into clusters in a user-friendly and transparent manner [34]. Ultimately, the docking results were examined and crosschecked using multiple tools including Autodock Vina and the BIOVIA Discovery Studio Visualizer [35]. However, the docking step for reference molecule was cautiously conducted through Autodock Vina 1.1.2. in this case, 5.63Å, 29.26Å and 22.13Å of X, Y and Z values respectively were defined for gridbox center coordination. Size in 43.73Å, 65.95Å and 70.66 Å of X, Y and Z respectively was considered for defining the gridbox dimensions. Moreover, the exhaustiveness values were selected in 9 which is default in AutoDock Vina. Docking of the reference molecule into the active site produced RMSD < 2.0 Å confirmed docking reliability followed by a rigid receptor mode.

### Toxicity analysis of the potential ligands

The potential VacA toxin inhibitors (ligands) were evaluated for their ADMET (Absorption, Distribution, Metabolism, Excretion, and Toxicity) properties using the pkCSM and SwissADME tools. The pkCSM ADMET test primarily evaluates various characteristics of potential ligands. The analysis covered several ADMET aspects, including absorption parameters like Caco-2 permeability, human intestinal absorption, water solubility, and interactions with P-glycoprotein; distribution factors such as human volume of distribution at steady state (VDss), blood-brain barrier permeability, fraction unbound in humans, and central nervous system (CNS) permeability; metabolism details involving inhibition of cytochrome P450 enzymes and substrates for CYP2D6 and CYP3A4; excretion characteristics including interactions with renal OCT2 and total clearance; as well as toxicity assessments encompassing rat LD50, tetrahymena pyriformis toxicity, AMES toxicity, minnow toxicity, maximum tolerated dose, hepatotoxicity, chronic oral toxicity in rats, skin sensitization, and hERG channel inhibition [36]. Numerous quick yet reliable prediction tools for assessing physicochemical characteristics, pharmacokinetics, drug resemblance, and adherence to medicinal chemistry principles are publicly available through SwissADME. These techniques involve proprietary inventions like BOILED-Egg, iLOGP, and Bioavailability Radar [37].

### Molecular dynamics (MD) simulation

The most favourable way in which the ligand-receptor complex bindings were assessed using a 100 nanoseconds (ns) molecular dynamics (MD) simulation, which served to anticipate the enduring stability of the complex within a defined system [38]. Molecular dynamics simulation was conducted within a system that included water molecules, and the temperature and pressure were maintained to closely mimic physiological environmental conditions. The simulation procedure was carried out using the GROMACS program (version 2024.4). In this case, the CHARMM27 force field with the TIP3P water model was applied, and a 1 nm diameter cubic edge was designed to perform the simulation. To neutralize the system, 0.15 M NaCl was used, and an appropriate number of ions were added accordingly. During the energy minimization process, simulations were conducted under constant number of particles, volume, and temperature (NVT) as well as constant number of particles, pressure, and temperature (NPT) conditions using the steepest descent algorithm, running for up to 50,000 steps over 100 picoseconds. In this case, the Parrinello-Rahman barrostate and V-rescale thermostate was independently applied accordingly. Finally, we employed a 100 ns MD simulation and collected the data every 2 fs of time step. All trajectories from the production of MD simulations were analyzed using GROMACS tools, including g_rms for root-mean-square deviation (RMSD), g_rmsf for root-mean-square fluctuation (RMSF), g_gyrate for radius of gyration (Rg), g_sas for solvent-accessible surface area (SASA), and g_hbond for the number of hydrogen bonds (hbond) [39].

### Principal component analysis and free energy landscape calculation

Principal Component Analysis (PCA) is a computationally intensive method that used to uncover the most important motion of complexes as well as conformational changes. This technique is helpful in MD simulation through dimensionality reduction, functional motion identification, and calculation of the primary component that contributes most of the system’s variability [40]. In this case, we adjusted all the trajectories obtained from the production of MD to minimize both rotational and translational motions. A covariance matrix was then computed to get the most abundant motions. Then, eigenvectors and eigenvalues were calculated by diagonalizing the matrix, and they were allowed to project onto the first two principal components (PC1 and PC2). 2D projection of eigenvalues and eigenvectors that depict the overall dancing motions of the complexes along different directions in multidimensional space were generated by utilizing g_covar and g_anaeig protocol from GROMACS package. Finally, the most significant movements of the complexes were mapped using principal eigenvectors to the Cartesian trajectory coordinates. Moreover, Free Energy Landscape (FEL) was computed to understand the most thermodynamics contribution to the conformational changes during the course of 100 ns simulation [41]. All the trajectories from production MD were subjected to analyze energy landscape (-ls) by using g_sham protocol of GROMACS package, maintaining the following formula -


$$\begin{array}{c} \Delta \text{G }=\;\sum -kBT\;ln\;(\text{PA}-\text{PB}) \end{array}$$

Where ΔG is denoted for Gibbs free energy and *T* represents the temperature. The Boltzmann constant is mentioned by *kB* and PA and PB corresponds to the probabilities of the likelihood of the complexes into two dynamic states in the course of the simulation.

### MM/GBSA binding energy calculation

To further quantify the binding affinity of the selected ligands toward the VacA p55 domain, Molecular Mechanics Generalized Born Surface Area (MM/GBSA) calculations were performed using trajectories obtained from the molecular dynamics simulations. This technique helps to estimate the binding free energy (ΔG_bind) and to decompose the energetic contributions governing ligand–protein interactions. The total binding free energy (ΔG_bind) was calculated according to the following equation:


$$\begin{array}{c} \Delta \text{G}\_\text{bind}=\text{G}\_\text{complex}-(\text{G}\_\text{protein}+\text{G}\_\text{ligand}) \end{array}$$

where G_complex, G_protein, and G_ligand represent the free energies of the protein–ligand complex, the isolated protein, and the ligand, respectively. In this case, last 20 ns (8000–10001 frames) were considered for the calculation as well as MM/GBSA profiling. Moreover, the total binding energy was further decomposed into individual energetic components, including van der Waals interactions (ΔE_vdW), electrostatic interactions (ΔE_elec), polar solvation energy (ΔG_GB), and non-polar solvation energy (ΔG_SA).

## Results

### Protein identification and quality assessments

Rapid Annotation using Subsystem Technology (RAST) provides high-quality genome annotations for the Prokaryotes across the whole phylogenetic tree and emphasizes the potential threat posed by these strains and underscores the significance of targeting virulence factors. RAST’s annotation capabilities enable the identification of genes linked to pathogenicity, providing researchers with a roadmap for prioritizing targets crucial for disease progression.

According to RAST annotation, all variants of *H. pylori* were supposed to produce VacA toxin by the gene *VacA* (S1 Table). Moreover, the RAST-Seed viewer analysis indicated that five strains of *H. pylori* (*H. pylori* puno135, *H. pylori* strain ATCC43504, *H. pylori* PMSS1, *H. pylori* Hp A-11, *H. pylori* FDAARGOS_299) were distributed in “Virulence, Disease and Defense” category and “Toxins and Superantigens” subcategory. Hence, the virulence factor VacA was selected as the target for the development of novel drugs against *H. pylori* infection.

The crystal structure of the VacA p55 domain was determined at a minimum Bragg spacing of d_min_ = 2.4 Å, using experimental phases obtained through multiple isomorphous replacement and anomalous scattering techniques. The p55 structure is mostly an alpha-helix with a right-handed parallel twist, but it also has a compact globular domain (residues 736–811) at the C-terminus that contains both alpha-helical and beta-sheet secondary structural elements. The alpha-helical “calf” stretches to a length of 65 Å, with widths varying from 25 to 31 Å. In contrast, the C-terminal “foot” measures roughly 17 x 24 x 43 Å. Within the crystal structure, p55 domains pair up by converging at their N-terminal strands, aligning along a 2-fold symmetry axis.

**Fig 2:**
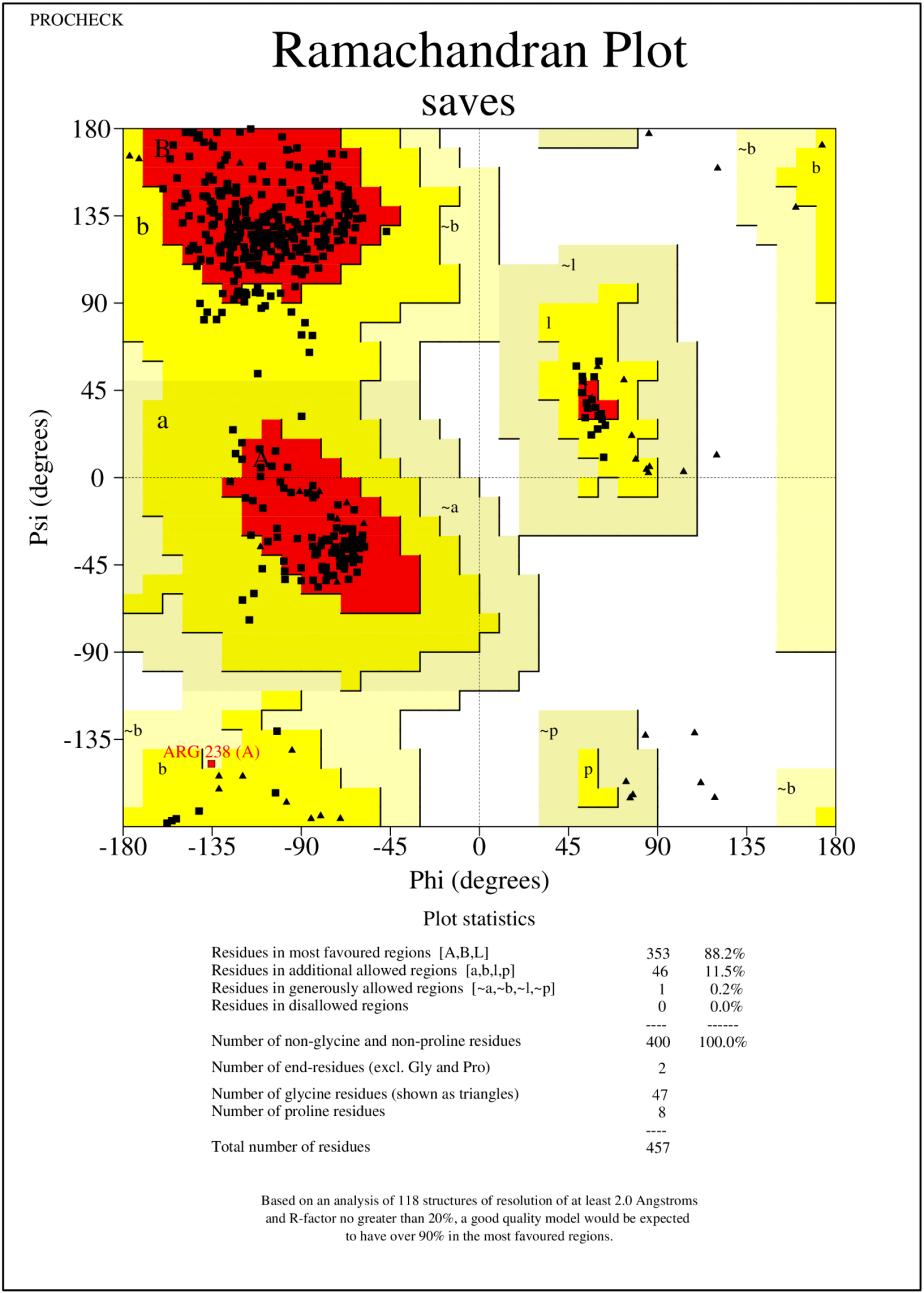
Ramachandran plot of refined modeled VacA toxin. Ramachandran plot uncovering refinement of the structure for recognizing permissible conformations of VacA protein.

**Fig 3:**
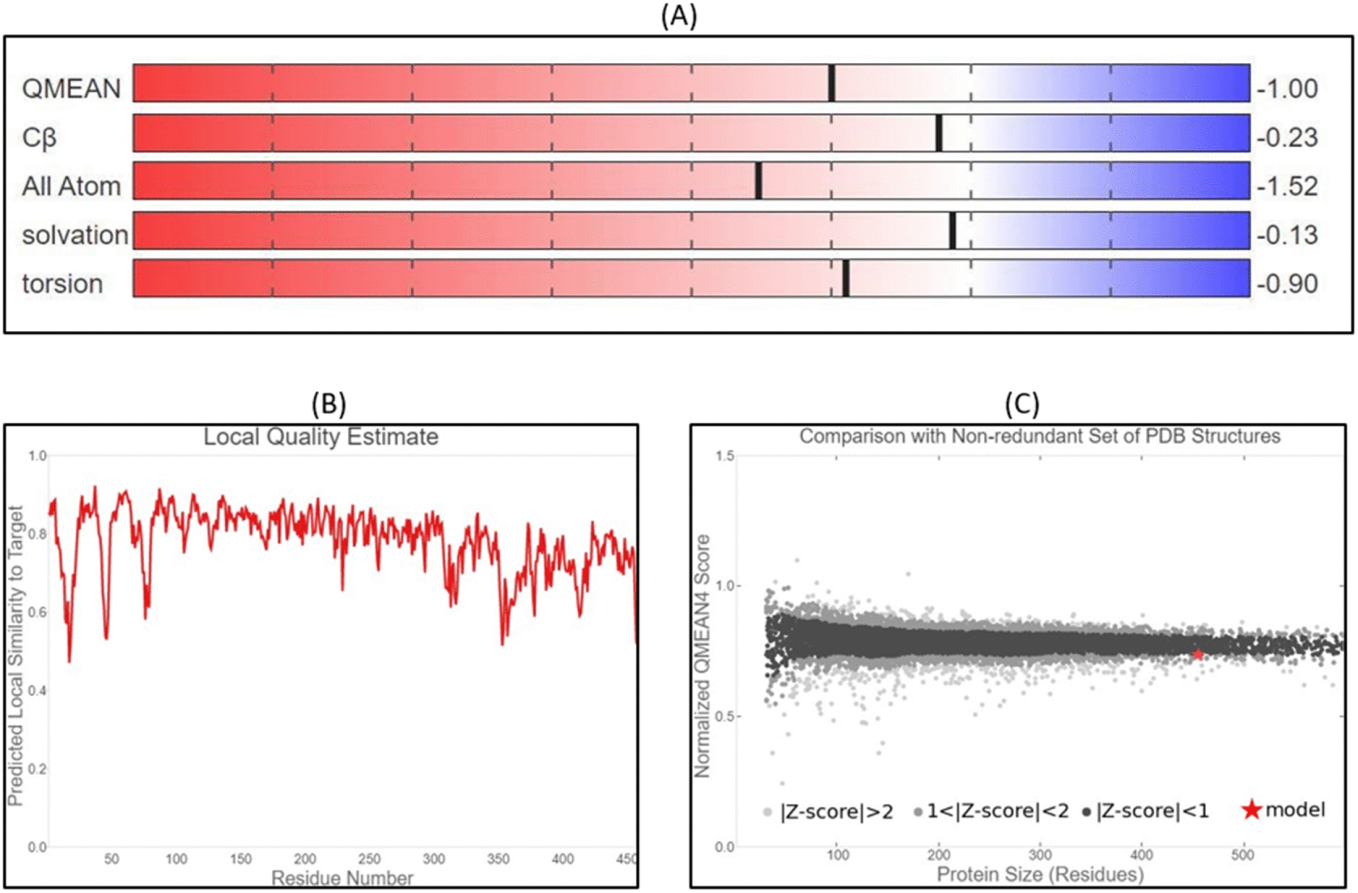
Overall quality assessments of the VacA protein structure. A) QMEAN value as well as individual Z-scores. B) The ProSA energy plot showing local model quality. C) Estimated absolute quality plot from QMEAN analysis.

To evaluate the refined homology model of the toxin protein, histograms with 4-degree intervals were used to record the frequency of Φ (Phi; C-N-CA-C) and Ψ (Psi; N-CA-C-N) angles across all analyzed groups. The contour lines are established based on the number of identified Φ/ Ψ pairs (Fig 2). The protein model, refined through Galaxy refiner, has shown that the molecules are in 98.68% favored region (Table 1). The protein’s Qualitative Model Energy Analysis (QMEAN) Z-score was determined to be −1.00, which indicated that the QMEAN score was predicted good (Fig 3A). QMEAN is a composite scoring function designed to evaluate the fundamental geometric features of protein structures. Moreover, four distinct components including local geometry agreement, all atom pairwise energy, solvation potential and torsion angle potential contributing to the overall QMEAN quality scores. The ‘white area’, shown in the bar plots, represents numerical values near zero, suggests that the refined model closely matches the characteristics typically found in experimental structures of a similar size (Fig 3A). The positive values imply that model scores are typically greater than those of experimental constructions. Conversely, negative values suggest that the model results, on average, fall below those of experimental structures. Meanwhile, the energy plot in Fig 3B illustrated the overall quality of the local model. The lowest standards in the energy plot are indicative of the nativity and stability of the residues, and in cases where a score below 0.6 is observed, it is expected that these residues will have low quality. The QMEAN Z-score of the model was calculated using the mean and standard deviation around the star’s (Red) location (Fig. 3B). Most importantly, the refined protein found a Z score of <1, which was considered good (Fig 3C). The refined structure achieved ideal scores for MolProbity (1.32), with zero Ramachandran and rotamer outliers, as well as zero bad bonds. Although the clash score and c-beta deviations slightly increased compared to the ideal values, they remain within an acceptable range (Table 1).

**Table 1:** Quality assessment of modeled VacA toxin protein of *H. pylori.*

Assessment criteria of the refined structure	Score of the refined structure	Ideal score
MolProbity Score	1.32	As low as possible (close to zero)
Clash Score	5.78	Zero
Ramachandran Favoured	98.68%	>98%
Ramachandran Outliers	0.00%	<0.2%
Rotamer Outliers	0.00%	<1%
C-Beta Deviations	7 (A220 TYR, A62 ASP, A193 LEU, A293 ARG, A236 ILE, A455 THR, A226 PHE)	Zero
Bad Bonds	0/ 3527	Zero

### Selection of active site and physiochemical properties

**Fig 4:**
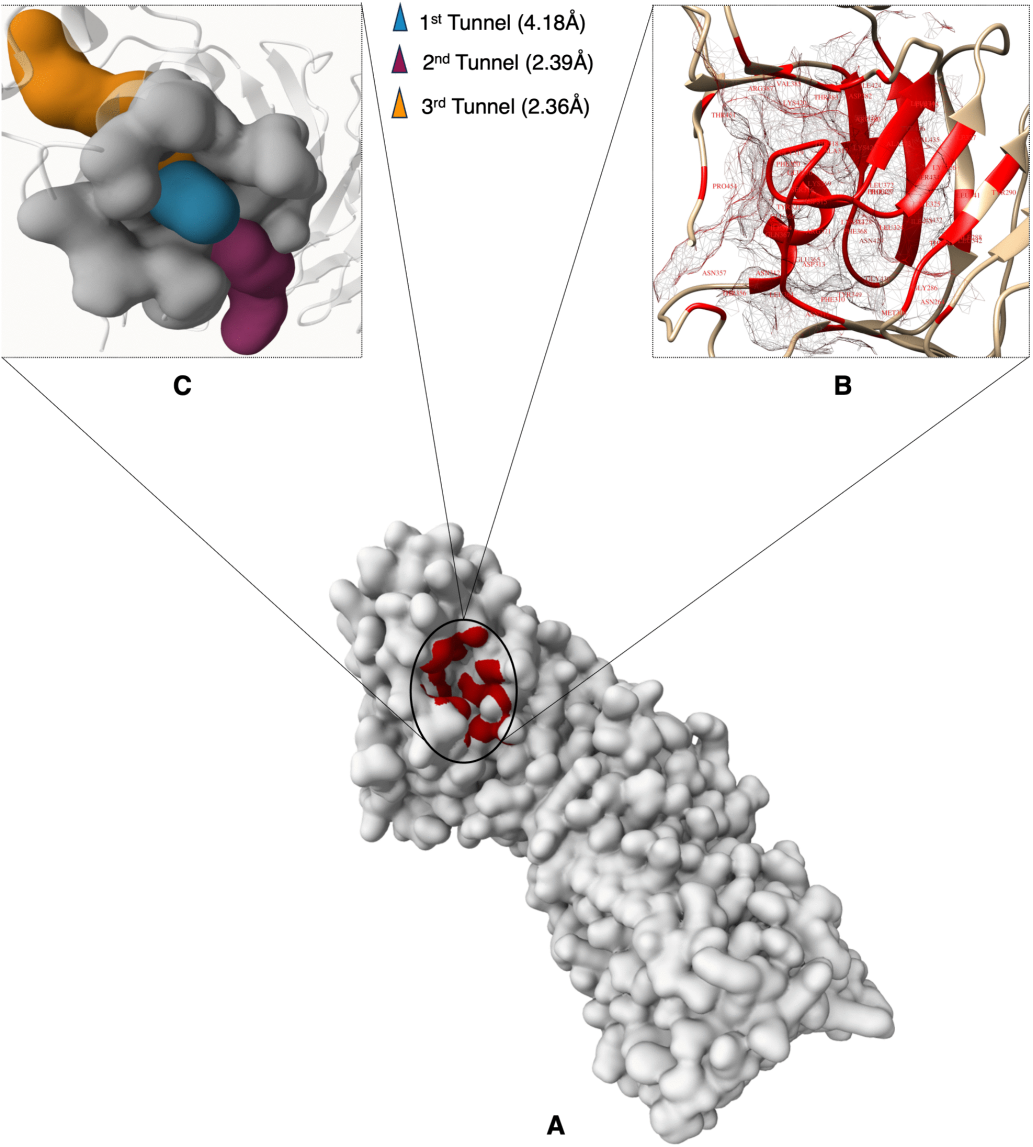
Structural characterization of the predicted binding pocket and internal tunnels within the VacA p55 domain. (A) Surface representation of the VacA p55 domain highlighting the predicted binding pocket (red). (B) Close-up view showing pocket residues and cavity architecture. (C) Internal tunnel network with three tunnels: Tunnel 1 (blue, 4.18 Å), Tunnel 2 (magenta, 2.39 Å), and Tunnel 3 (orange, 2.36 Å), indicating potential ligand access pathways.

An active site of a protein offers the specific, often deeply buried binding pocket where catalysis occurs. In this case, the active sites were predicted by using two different software i.e. CASTp 3.0 and PrankWeb on the p55 domain of VacA toxin in order to find the physiochemical properties of the pocket. Total surface area and the volume of the pocket were 203.064 (SA) Å^2^ and 131.268 (SA) Å3, respectively. A single pocket was found in CASTp server and the location of the key amino acid residues in the active site were identified as THR^287^, ASP^315^, ALA^317^, THR^318^, PHE^320^, TYR^321^, LYS^322^, PRO^323^, LYS^326^, TYR^375^, ASN^378^, ASN^379^, ARG^380^, THR^383^, CYS^384^, VAL^385^, VAL^386^, ARG^387^, ASP^391^, ALA^394^, CYS^395^, and ALA^398^. However, seven distinguished pockets were identified through PrankWeb tool (Table 2), where both of the tools predicted the similar position for pocket1. These additional pockets may facilitate allosteric site that could be classified as either inhibitory, activating or regulatory site enabling proper function of the protein. Moreover, these properties may enhance the involvement of the complex metabolic consequences. Meanwhile, tunnel information also explored to investigate the view of the entry channel of the p55 domain for penetration of ligand where three sufficiently spaced tunnel (4.18Å, 2.39 Å and 2.36 Å) were inspected (Fig 4). Hence, sufficient tunnels for the penetration of the ligand molecule were accommodate into VacA p55 domain.

**Table 2:** Key physiochemical and geometrical features provide insight into pocket accessibility and its suitability for small-molecule ligand binding.

Alias	Rank	Score	Probability^*^	SAS Points	Surf Atoms	Center Coordination (Å)			Residues Identifications
						X	Y	Z	
Pocket1	1	6.79	0.347	63	32	316.34	147.39	235.53	Asp^315^, Ala^317^, Thr^318^ Pro^323^, Lys^326^, Tyr^375^, Asn378, Asn^379^, Arg^380^, Thr^383^, Val^385^, Arg^387^, Asp^391^, Ala^394^, Cys^395^
Pocket2	2	2.44	0.066	40	27	321.75	159.50	241.71	Phe^320^, Tyr^321^, Thr^356^, Asn^357^, Val^362^, Glu^366^, Gln^367^, Glu^370^, Thr^451^, Asn^452^, Leu^453^, Pro^454^
Pocket3	3	2.23	0.054	36	11	265.76	173.93	211.48	Asn^109^, Lys^111^, Lys^22^, Thr^49^, Ser^80^, Asn^82^, Glu^84^
Pocket4	4	2.1	0.048	27	15	279.99	159.53	205.11	Arg^106^, Gly^125^, Asn^126^, Gly^127^, Phe^129^, Ala^149^, Thr^167^, Asn^168^, Ser^171^, Glu^174^
Pocket5	5	1.91	0.039	28	13	326.86	161.58	229.69	Glu^366^, Lys^369^, Lys^420^, Ile^424^ Ser^425^, Lys^426^, Thr^427^
Pocket6	6	1.5	0.021	24	14	297.93	166.17	233.53	Glu^235^, Thr^237^, Thr^260^, Gly^262^, Gln^263^, Asn^284^
Pocket7	7	0.81	0.003	5	7	318.68	156.87	226.56	Ile^327^, Leu^372^, Ala^373^, Leu^374^, Val^435^

* Higher probability means higher prediction accuracy.

### Ligand preparation and selection of a reference drug

From the ready-to-dock ligand database ZINC15, 3000 ligands were downloaded, and several libraries were created according to their molecular weights. Among them, 178 ligands were found in the active site-bound protein-ligand complex (see S2 Table for the list of active site-bound ligands and their binding affinity). Finally, eight potential ligand molecules (see S4 Fig for their chemical structure) were selected for further study, considering their higher binding affinity and ADMET properties. They adhered to the “Lipinski Rule of Five,” as all ligands satisfied the criteria of having a molecular weight under 500 g/mol, fewer than five hydrogen bond donors, less than 10 hydrogen bond acceptors, high lipophilicity (LogP < 5), and a molar refractivity between 40 and 130 (S3 Table). From the available drugs in the market, a widely used medication named Ranitidine, which is primarily used to treat gastric or ulcer-related conditions such as indigestion, heartburn, acid reflux, and gastroesophageal reflux disease, was selected to compare the potential efficacy and performance of our proposed ligand molecules.

### Molecular docking of ligands and reference molecule

**Fig 5:**
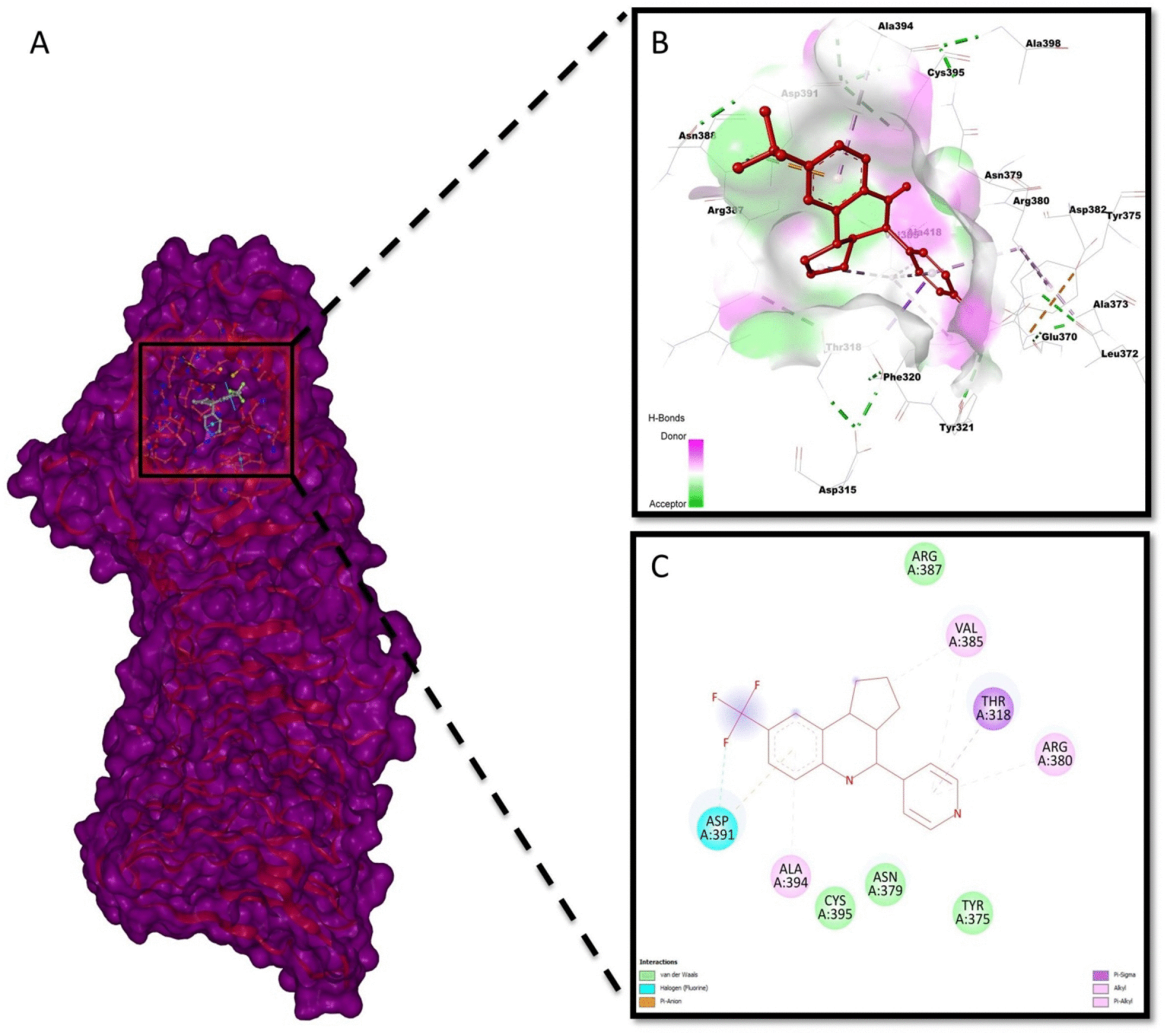
Molecular docking of the ligand 8 with the p55 domain of VacA toxin. A) 3D view of the docked complex. B) Interactions of the ligand 8 with the residues from VacA toxin covering 5Å of pocket area. C) 2D depiction of the surrounding amino acids interacted with the ligand 8.

**Fig 6:**
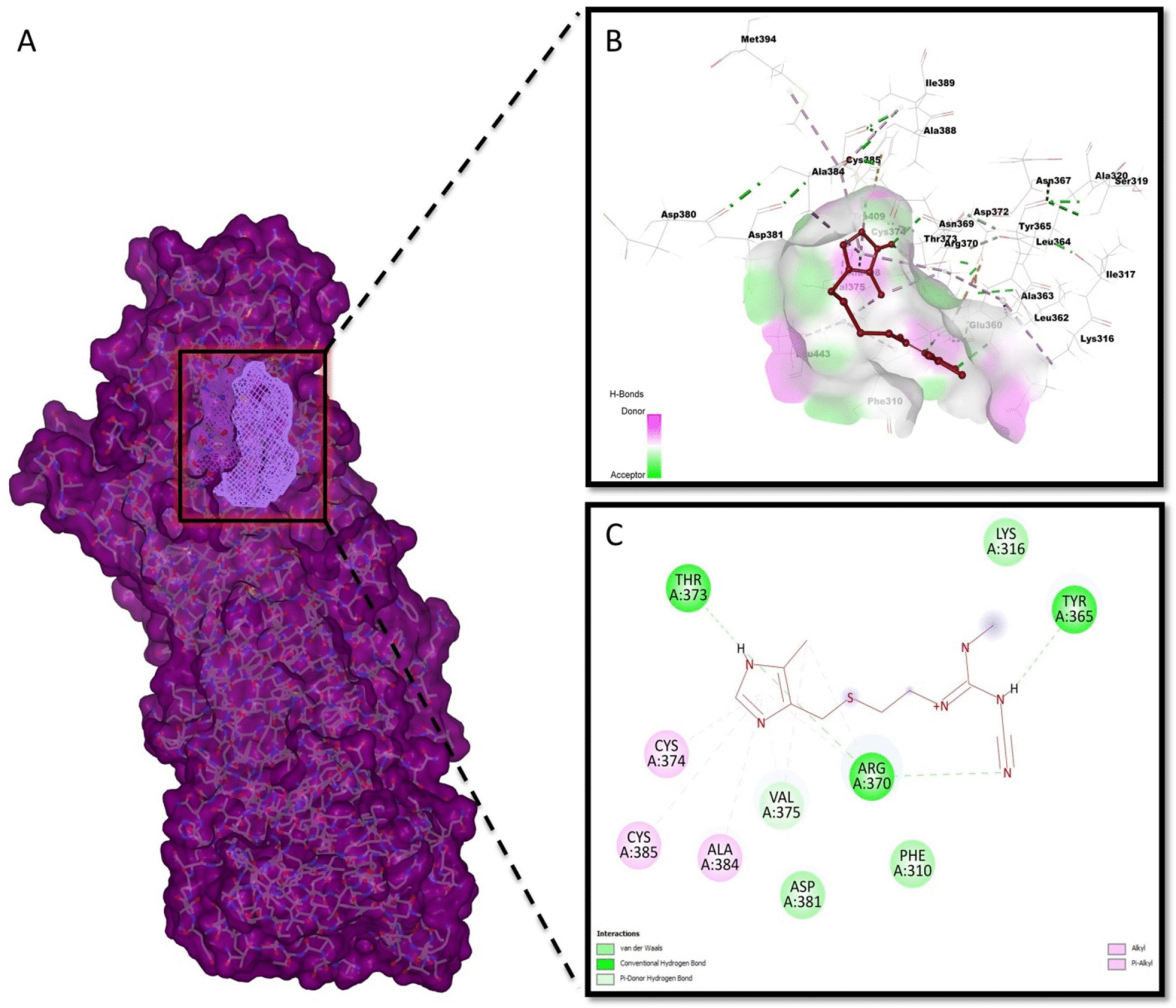
Molecular docking of the Ranitidine (reference molecule) with the p55 domain of VacA toxin. A) 3D view of the docked complex. B) Interactions of the Ranitidine with the residues from VacA toxin covering 5Å of pocket area. C) 2D depiction of the surrounding amino acid interacted with the Ranitidine.

Through rigorous analysis encompassing both binding affinity and ADMET prediction for toxicity in the human body, eight ligands emerged as promising inhibitors. These eight selected potential ligand molecules, the ligands ZINC9086561 (designated as ligand 1), ZINC41084323 (ligand 2), ZINC408534388 (ligand 3), ZINC8665141 (ligand 4), ZINC1379186 (ligand 5), ZINC8821620 (ligand 6), ZINC3644748 (ligand 7), ZINC4004291 (ligand 8) showed strong binding affinity (−8.2 kcal/mol, −7.8 kcal/mol, −7.8 kcal/mol, −7.3 kcal/mol, −7.3 kcal/mol, −7.2 kcal/mol, −7 kcal/mol and −6.9 kcal/mol respectively) with the p55 domain of VacA toxin (Table 3). The interaction between each of these eight ligand molecules and VacA toxin was analyzed (S5 and S6 Figs). Molecular docking showed that the ligand 8 (lead molecule) interacts well with VacA toxin (Fig 5). The amino acids, THR^318^, ARG^380^, Val^385^, ASP^391^, ALA^394^ and CYS^395^, of VacA toxin interact with ligand 8 (Fig 5). The molecular docking interaction between the control molecule ranitidine and VacA showed a low-affinity score of −5.0 kcal/mol (Fig 6 and Table 3). The amino acids of the p55 domain of VacA toxin that interact with the ligand and control molecules and their bond types are included in Table 3. ARG^380^ and VAL^385^ are the most common amino acids that interact with all the ligands except ligand 2, while they showed no interaction with ranitidine. Thus, the docking results suggest strong predicted binding interactions for ligand 8.

**Table 3:** Essential information of the potential ligands and their binding affinity during interaction with the p55 domain of VacA toxin.

Ligand (ZINC ID)	Docking Score (kcal/mol) (Best Range)	Key Interacting Residues	Avg. RMSD (nm) (Equilibrated Window)	ADMET Profile (Key Predictions)	Synthetic Accessibility Score*	Lipinski Compliance	Bond Status
					(1–10)		
ZINC9086561	−8.2	Thr^318^, Lys^326^, Tyr^375^, Arg^380^, Val^385^, Asp^391^	0.28 ± 0.03	High GI absorption; BBB moderate; AMES positive; predicted hepatotoxicity	3.8	Yes	Pi-Donor Hydrogen Bond, Carbon Hydrogen Bond Conventional Hydrogen Bond
	(−8.2 to −7.4)						
ZINC41084323	−7.8	Lys^239^, Ala^241^, Val^266^, Asp^268^, Asn^328^, Asn^377^, Ser^329^	0.26 ± 0.02	High GI absorption; non-AMES; hepatotoxicity predicted	3.9	Yes	Alkyl/ Pi-Alkyl, Pi- Sigma, Conventional Hydrogen Bond, Carbon Hydrogen Bond
	(−7.8 to −7.1)						
ZINC408534388	−7.8	Val^385^, Arg^380^, Arg^387^, Asp^391^, Ala^394^, Asn^378^	0.27 ± 0.03	Moderate GI absorption; AMES positive; non-hepatotoxic	4.1	Yes	Conventional Hydrogen Bond, Alkyl/ Pi-Alkyl, Pi-Anion, Pi- Sigma
	(−7.8 to −7.2)						
ZINC8665141	−7.3	Arg^380^, Val^385^, Asn^378^, Asn^379^ Lys^326^, Tyr^375^	0.27 ± 0.02	High GI absorption; non-AMES; hepatotoxicity predicted	3.6	Yes	Pi-Alkyl/ Pi-Pi T-Shaped, Conventional Hydrogen Bond, Pi-Cation,
	(−7.3 to −6.9)						
ZINC1379186	−7.3	Ser^329^, Tyr^375^, Cys^395^, Val^385^, Arg^380^	0.26 ± 0.03	High GI absorption; non-AMES; hepatotoxicity predicted	3.7	Yes	Conventional Hydrogen Bond, Alkyl/ Pi-Alkyl, Pi-Sulfur,
	(−7.3 to −6.8)						
ZINC8821620	−7.2	Thr^287^, Pro^323^, Asp^325^, Lys^326^, Tyr^275^, Arg^380^, Val^385^, Cys^395^	0.27 ± 0.02	High GI absorption; non-AMES; non-hepatotoxic	3.5	Yes	Carbon-Hydrogen Bond, Pi Anion/Pi-Cation, Alkyl, Conventional Hydrogen Bond
	(−7.2 to −6.7)						
ZINC3644748	−7.0	Asn^378^, Thr^383^, Val^385^, Arg^380^, Ala^395^	0.25 ± 0.02	High GI absorption; non-AMES; non-hepatotoxic	3.2	Yes	Conventional Hydrogen Bond, Halogen (Fluorine), Alkyl/ Pi-Alkyl
	(−7.0 to −6.6)						
ZINC4004291	−6.9	Cys^395^, Val^385^, Asp^391^, Ala^394^, Thr^218^, Arg^380^	0.24 ± 0.02	High GI absorption; non-AMES; non-hepatotoxic; favorable ADMET profile	3.1	Yes	Pi Anion/Pi Sulfur, Alkyl/ Pi-Alkyl, Halogen (Fluorine), Pi- Sigma
	(−6.9 to −6.5)						

* Lower scores indicate easier synthetic feasibility.

### Pharmacological properties and Toxicity prediction

All eight ligands and reference molecules were subjected to toxicity and other pharmacokinetic test, including optimal solubility, high Caco permeability, excellent human intestinal absorption, little skin permeability, uniformly distributed volume of distribution, and positive blood-brain barrier (BBB) and central nervous system (CNS) penetration values in the pkCSM server, and SWISS ADMET (S4–S12 Tables).

ADMET test of ligand-8 showed it has optimal water solubility of −5.576 log mol/L, low amount of Caco permeability 1.69 log Papp in 10 cm/s, Human intestinal absorption (HIA) 91.688, skin permeability −2.687 log Kp, volume of distribution at steady state (VDss) value 0.521 log L/kg, BBB(+) permeability value −0.125, CNS(+) permeability value −1.421, total clearance of 0.024 log ml/min/kg (S11 Table). The substance is not expected to inhibit hERG I or II and has no AMES toxicity. It has no hepatotoxicity and skin sensitization potential. The maximum tolerated dose in humans was recorded as –0.706 log mg/kg/day. Oral acute toxicity (LD50) in rats was 3.069 mol/kg, while the chronic toxicity (LOAEL) was measured at 0.853 log mg/kg/day. Predicted environmental toxicity values fell within acceptable ranges, with *Tetrahymena pyriformis* toxicity at 1.828 log µg/L and minnow toxicity at 1.22 log mM. The ADMET profile of the reference compound, Ranitidine, included water solubility of −3.299 log mol/L, Caco-2 permeability 1.239 log Papp (10^-6 cm/s), human intestinal absorption (HIA) of 90.919%, skin permeability −2.971 log Kp, evenly distributed VDss of 0.128 log L/kg, BBB+ permeability at −0.769, CNS+ permeability at −3.092, and total clearance of 0.893 log ml/min/kg (S12 Table).

In toxicity analysis, Ranitidine also did not exhibit any AMES toxicity or hepatotoxicity and was not an hERG I/ hERG II inhibitor. The maximum tolerated dose in humans was 0.139 log mg/kg/day. The oral acute toxicity (LD50) for rats was 2.674 mol/kg, while the chronic toxicity (LOAEL) was 1.144 log mg/kg body weight per day. However, the compound showed positive skin sensitization. Environmental toxicity values comprised *T. pyriformis* toxicity at 0.437 log µg/L and minnow toxicity at 1.499 log mM. These profiles explore the predicted pharmacokinetic and toxicological properties of the compounds based on in silico ADMET analysis. Among the eight potential ligand molecules, Ligand 8 showed the least amount of toxicity compared to the other seven candidate ligands (S11 Table) and the control molecule, Ranitidine (S12 Table). Hence, Ligand 8 was selected as lead molecule for further studies.

### Molecular dynamics simulation

**Fig 7:**
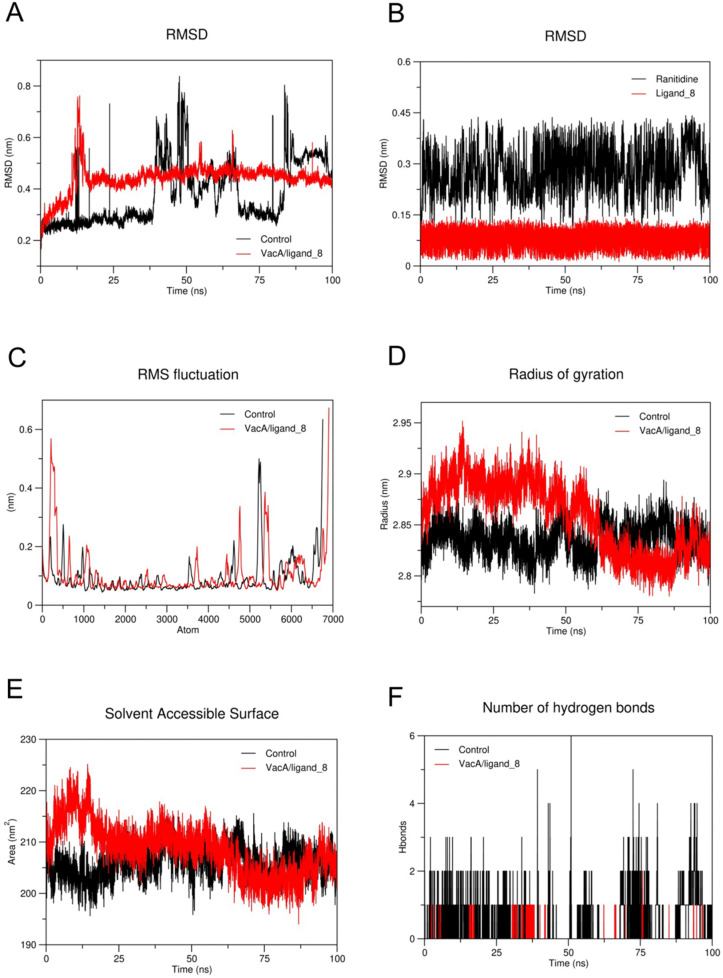
Molecular Dynamic (MD) simulation of both VacA/Ranitidine (control) and VacA/ligand 8 (leading) complexes. A) Root Mean-Square Deviation (RMSD) plots of VacA-ligand 8 complex. B) RMSD plots of ranitidine and ligand 8 molecules. C) Root Mean-Square Fluctuation (RMSF) plots of VacA/Ranitidine (control) and VacA/ligand 8 complexes. D) Radius of Gyration (Rg) plots of VacA/Ranitidine and VacA/ligand 8 complexes. E) Solvent Accessible Surface Area (SASA) plots of VacA/Ranitidine and VacA/ligand 8 complexes. F) Plots estimating the number of hydrogen bonds of VacA/Ranitidine (control) and VacA/ligand 8 complexes.

Molecular dynamics (MD) simulation serves to validate the strength of protein-ligand complexes within a defined and controlled environment. The RMSD of a period of 100 nanosecond has been determined for the selected compound, Ligand 8 (lead compound), and a compound (Ranitidine) acted as a reference molecule for comparing the overall stability of the Ligand 8-VacA complex (leading complex). Accordingly, both the compounds (ligand 8 and Ranitidine) were considered to analyse their stability (ligand RMSD) during docking. The RMSD results indicated the stability of the interactions involving the target lead compound (Ligand 8) to the p55 domain of VacA compared to the reference compound. In this case, two RMSD profiles demonstrate a distinguished pattern of fluctuations where VacA/ligand 8 complex experienced mostly stable from 15.5 Å to the end of the simulation compared to the reference compound (Fig 7A and 7B). Ranitidine, used as the reference molecule, exhibited higher RMSD fluctuations with an average of 2.977 nm, whereas ligand 8, considered a potential new drug, showed a much lower average RMSD of 0.075 nm. A lower RMSD indicated that the ligand 8 was found more stable than the reference molecule. Furthermore, the analysis of the RMSF plots examines the flexibility of the residues within the p55 domain of the VacA toxin during complex formation while comparing to the same receptor with a reference molecule. In this case, overall fluctuations in atoms of the VacA/ligand 8 leading complex were found mostly equivalent compared to control (VacA/ranitidine), however, the initial and final portion of the new complex get slightly higher fluctuation, suggesting to bear more flexible regions in both N termini and C termini. (Fig 7C).

For determining the solute’s center of mass, a crucial measurement of distribution of mass within a solute molecule, the radius of gyration (Rg) was calculated. The computed average Rg for the docking complex of the p55 domain of VacA and ligand 8 exhibited a declined fashion of fluctuation especially after 37.5 ns. After 49.6 ns, it has been taken down the control and followed up to the end of the simulation, resulting in compactness with proper folding over the simulation period (Fig 7D). Moreover, the solute’s surface area is made up of all the spots that the water probe can access when rolling over the solute, including its center. From Fig 7E, it was found that the SASA score of the docking complex (p55 domain of VacA and ligand 8) was significantly lower than its initial position. Interestingly, it was plateaued and mimicked some portion of the trajectory where the reference molecule exhibited similarly meeting together except very begining stage of simulation. This finding indicated that ligand 8 was deeply buried in the pocket site, contributing to a stronger binding affinity during molecular dynamics compared to the reference molecule, Ranitidine. Consequently, the SASA value also supports the Rg result as it helps to conclude that the VacA/ligand 8 complex is more compact and stable compared to the VacA/Ranitidine complex. Computation of the H-bonds can explore the important insightful information about the stability and dynamics of the protein-ligand interaction. The quantity of intermolecular hydrogen bonds inside the solute was observed during the 100 ns simulation, as shown in Fig 7F. In this case, a higher degree of hydrogens were exposed in the reference complex than the leading complex (VacA-ligand 8). Most of the hydrogen bonds are densely populated between the range of 0 and 2 Hbonds (Fig 7F) during the complex formation of the reference molecule and the VacA protein, suggesting higher strength and possible flexibility of the receptor to the reference molecule. However, in the VacA-ligand 8 complex, fewer hydrogen bonds were observed in this range of Hbonds compared to the control, which indicates the lower interaction due to less binding site accessibility or maybe due to the rigidity properties of the receptor protein towards ligand 8. Therefore, the overall MD simulations suggest stable structural behaviour of the complexes within the simulation timeframe.

### Principal component analysis and the Gibbs free energy landscape

**Fig 8:**
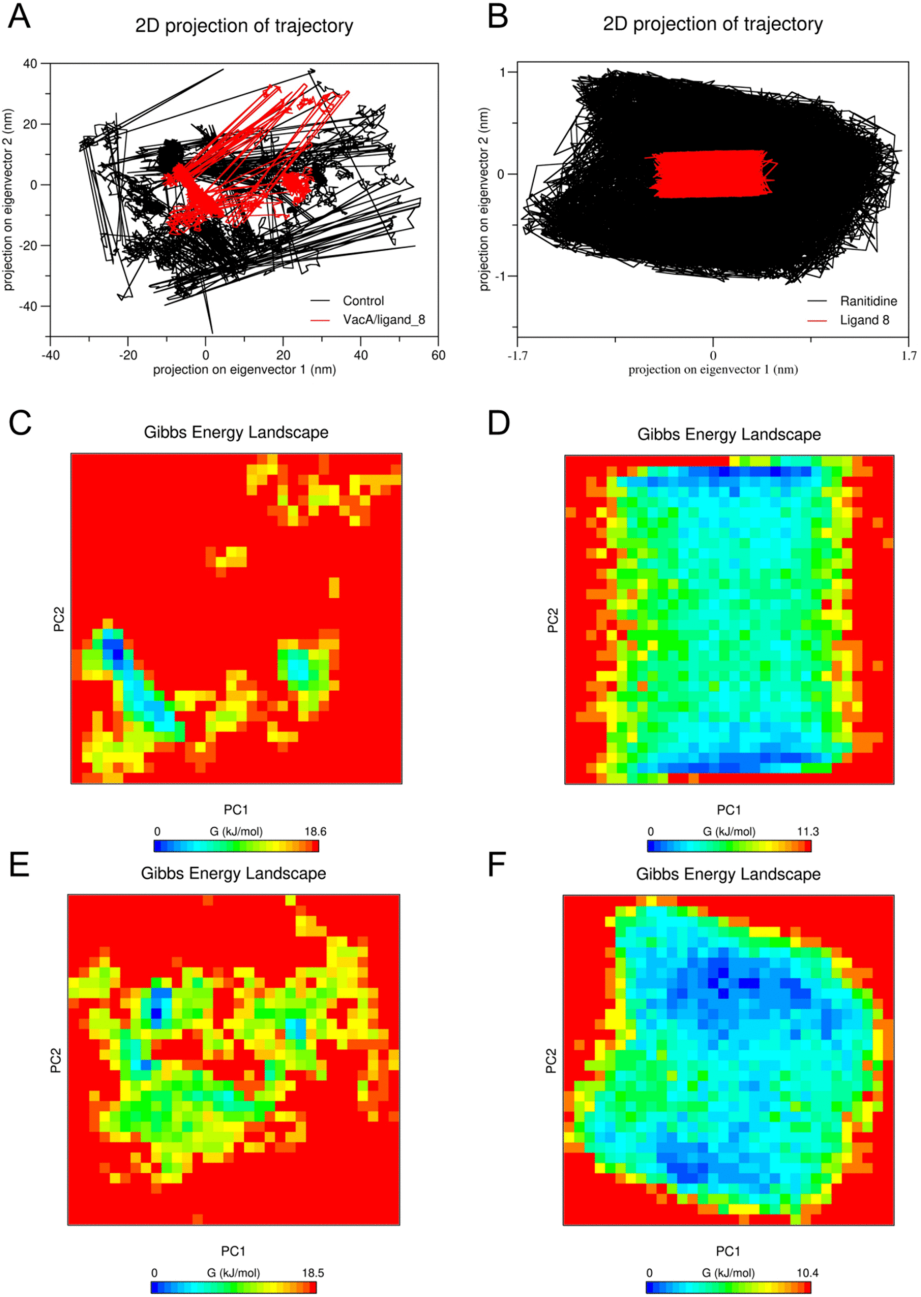
Principal Component Analysis (PCA) and Free Energy of Landscape (FEL) of both control (VacA/Ranitidine) and leading (VacA/ligand 8) complexes. A) 2D projection of the principal components along each direction of complex dynamics. B) 2D projection of the principal components along each direction of the reference (Ranitidine) and lead compound (ligand 8) dynamics. C) Gibbs free energy landscape of the leading (VacA/ligand 8) complex. D) Gibbs free energy landscape of the lead (ligand 8) compound. E) Gibbs free energy landscape of the control (Ranitidine/VacA) complex. F) Gibbs free energy landscape of the reference molecule (Ranitidine).

Principal component analysis explores the most significant movement of the complexes along the direction on a multidimensional space. According to 2D projection of the most significant eigenvectors of both complexes (VacA/ligand 8 and VacA/ranitidine control) and single lead molecule (ligand 8) with corresponding control (ranitidine) represented in the Fig 8A and 8B, respectively. Here, we traced the area of 714.84 nm^2^ and 315.36 nm^2^ of the covariance after diagonalizing the matrix for ranitidine/VacA toxin complex (control) and ligand 8/VacA toxin (leading complex), respectively. Similarly, 1.90 nm^2^ and 0.10 nm^2^ were the areas of covariance after diagonalizing the matrix identified for ranitidine and the ligand 8 molecule, respectively. These findings suggested that the leading complex has a comparatively smaller area of covariance where most of the eigenvectors produce significantly smaller as well as densed area according to Cartesian coordinations (Fig 8A and 8B). Furthermore, Gibbs free energy was calculated to understand the thermodynamic behavior during the simulation period, as shown in Fig 8C–8F. In this case, a very small variation of the Gibbs energy (ΔG) was observed between the leading complex and the control, having 18.6 KJ/mol and 18.5 KJ/mol, respectively (Figs 8C and 8E). Moreover, the leading molecule (ligand 8) and the ranitidine (reference) separately experienced very little differences in the Gibbs energy (ΔG) where 11.3 KJ/mol and 10.4 KJ/mol anticipated for the leading molecule and the reference, respectively (Fig 8D and 8F). These results revealed a very small deviation in the thermodynamic stability between the leading complex and the control.

### MM/GBSA binding free energy analysis

**Fig 9:**
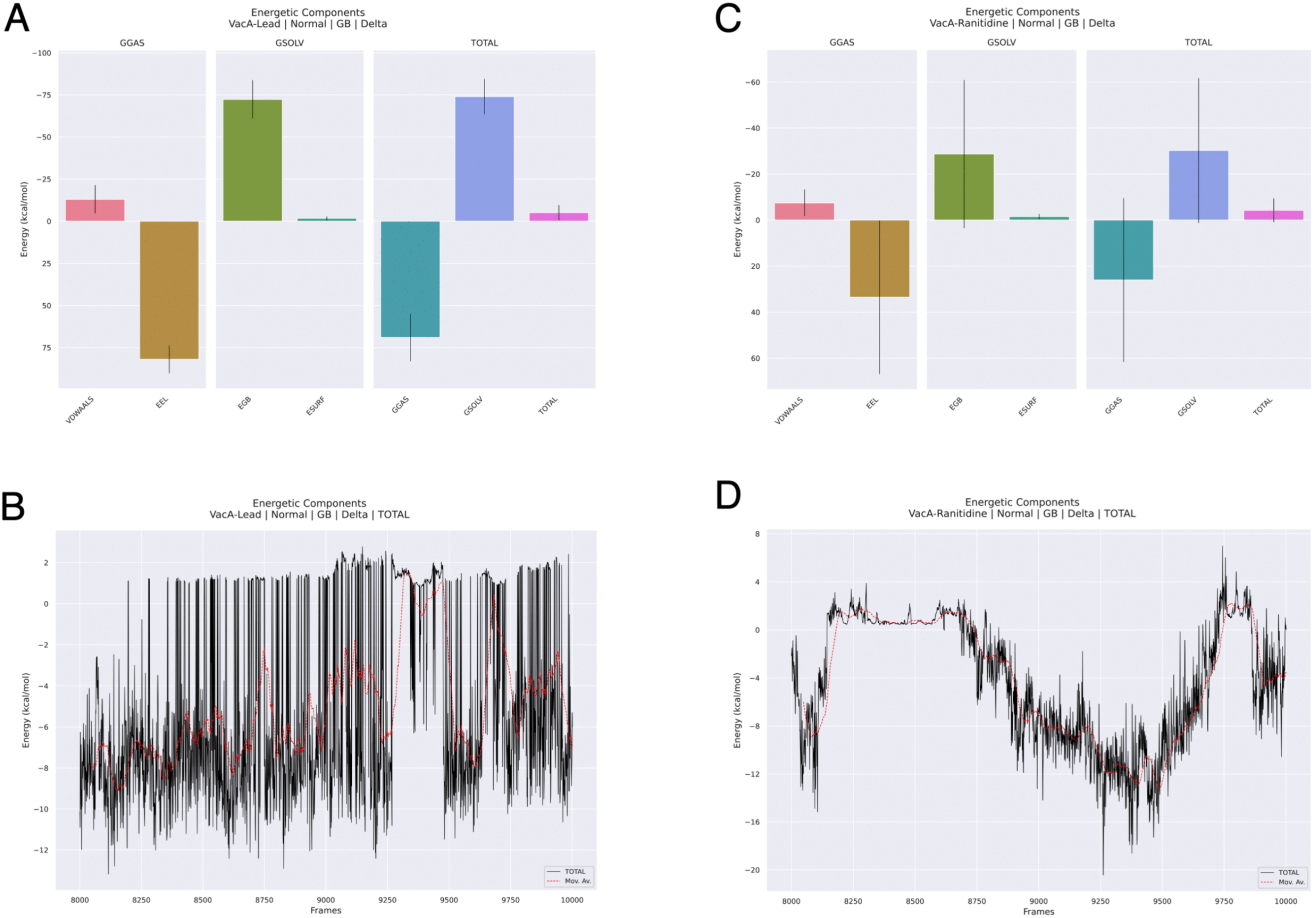
Comparative MM/GBSA binding free energy analysis of VacA–lead compound and VacA–Ranitidine complexes from last 20 ns of MD simulation. Panels A and C show the decomposition of binding free energy components**,** including van der Waals, electrostatic, polar solvation, and non-polar solvation contributions to the total binding free energy. Negative energy values represent favourable interactions contributing to complex stability. Panels B and D illustrate the time-resolved MM/GBSA binding free energy profiles obtained from MD simulation trajectories. The black curves represent instantaneous binding free energy values across simulation frames, while the red line indicates the averaged energy trend.

MM/GBSA binding free energy calculations were carried out utilizing snapshots taken from the equilibrated area of the MD trajectories in order to assess the energetic stability of ligand binding to the VacA p55 domain. For the ligand-protein complexes, the study produced time -resolved binding free energy profiles as well as energy component decomposition. Van-dar-Waals and electrostatic interactions contributed significantly to the total binding energy for the identified lead compound-VacA complex, according to the MM/GBSA energy decomposition (Fig 9A and 9B). This finding suggests that hydrophobic contacts and electrostatic interactions were crucial for stabilising the ligand within the binding pocket. The normal desolvation penalty linked to ligand binding was reflected in the polar solvation energy’s positive contribution. Throughout the simulation, the time-dependent binding free energy profile showed comparatively steady oscillations, resulting, a stable ligand engagement was established within the VacA pocket of p55 domain.

In contrast, the time-resolved binding free energy profile showed more variations and relatively fewer positive energetic contributions in the control complex containing ranitidine (Fig 9C and 9D). The overall energy balance seemed less stable than that of the discovered lead compound, despite the fact that van der Waals interactions also helped to stabilise this complex. Weaker or lesser consistent interactions between the control molecule and the VacA p55 binding region during the simulation are suggested by the greater variability in binding energy values.

Overall, the MM/GBSA results estimate the docking and molecular dynamics data by showing that the identified lead compound exhibits relatively stronger and more stable predicted binding interactions with the VacA p55 domain than the reference drug Ranitidine. Nevertheless, these results are still computational predictions that need to be verified experimentally.

## Discussion

*H. pylori* usually causes stomach infections by employing the virulence factor they express. This study focused on targeting the *H. pylori* infection by inhibiting its virulence factor, the vacuolating cytotoxin A (VacA), known to cause cancer and severe stomach damage in humans. VacA is a major virulence factor of *H. pylori* that contributes to gastric epithelial cell damage through receptor binding, oligomerization, and membrane pore formation. Structurally, VacA consists of the p55 domain responsible for host-cell receptor interaction. Following secretion, VacA monomers assemble into oligomeric complexes that insert into host cell membranes to form anion-selective pores, leading to cellular vacuolation and cytotoxic effects [17,18,42]. In this context, ligand binding within the predicted pocket may influence structural transitions required for VacA oligomerization or pore formation. Thus, the ligand occupancy within this pocket may interfere with VacA structural assembly or host receptor engagement, although this hypothesis requires experimental validation. Therefore, the p55 domain of the VacA toxin was rationally chosen as the target protein for molecular docking with various chemical compounds from the ZINC15 database to identify potential inhibitors of the VacA toxin.

In this study, the refined homology-modelled VacA toxin protein underwent rigorous assessment, anticipating structural reliability for subsequent analyses. Evaluation with the SWISS-MODEL Structure Assessment tool and Galaxy refiner revealed high occupancy in the favored region (98.68%) of the Ramachandran plot and a favorable Qualitative Model Energy Analysis (QMEAN) Z-score (−1.00), indicating robust structural integrity. Additionally, the refined structure exhibited ideal scores for MolProbity (1.32), Ramachandran outliers, rotamer outliers, and bad bonds. Despite minor deviations in clash score and c-beta deviations, the overall structure adhered to ideal scores, confirming its consistency for subsequent studies.

Ligands sourced from the ZINC15 database underwent stringent selection criteria to identify potential drug candidates targeting the p55 domain of *H. pylori* VacA toxin. Following the values of 3D ready-to-dock tranches, ligands with neutral pH values were downloaded and organized into libraries based on a molecular weight cutoff of 3000. Selection criteria prioritized ligands with higher binding scores and lower toxicity in ADMET predictions. Additional information regarding pocket associated amino acid identification help to understand the overall scenario of the pockets. In this case, seven different pockets were identified into different location of VacA toxin (Table 2). However, pocket1 among 7 pockets found similar to the pocket identified from CASTp, hence, crosscheck was maintained to confirm the exact site where ligand might bind with. Moreover, sufficient tunnel (4.18 Å, 2.39 Å and 2.36 Å) might facilitated successful entry of ligands to the p55 domain.

Molecular docking was performed using AutoDock Vina through PyRx, utilizing a flexible docking approach to investigate interactions between the ligand and the protein. Among the 3,000 ligands screened, 178 ligands (5.93%) were identified as binding to the active site of the p55 domain having optimum score ranging from −5.5 to −8.2 (S2 Table). With the help of additional downstream processes, eight ligands identified having optimum binding affinity and lower toxicity. These results indicate favourable binding energetics that support further investigation of the compound.

All eight ligands were subjected for rigorous ADMET testing to assess their pharmacokinetic and toxicological properties. Among these ligands, ligand-8 (lead compound ZINC4004291) exhibited favourable ADMET properties and low toxicity profiles. Ligand-8 demonstrated minimal toxicity, with no AMES toxicity, which could be a non-mutagenic agent. It does not inhibit hERG I or II, which suggests its lower risk of cardiotoxicity. It also does not show any hepatotoxicity or skin sensitization profiles. On the contrary, the toxicity profiles of the reference molecule, Ranitidine, exhibited positive skin sensitization, which may indicate potential dermatological concern. Along with the ADMET analysis, ligand-8 exhibited a high binding affinity of −7.0 kcal/mol to the active site cavity of the p55 domain of VacA toxin (Fig 5), comparing the reference compound which showed a weaker binding affinity of −5.0 kcal/mol (Fig 6). Due to its favourable pharmacological characteristics and low toxicity, ligand-8 is potentially distinguished as a promising drug candidate for treating *H. pylori* infection although experimental validation is required.

Moreover, molecular dynamics (MD) simulation is used to uncover the stability of protein-ligand complexes under defined and controlled conditions. In this study, a 100 ns MD simulation was performed to evaluate the long-term behavior and structural stability of the protein-ligand interactions. The outcomes from the MD simulation have been elucidated through metrics such as RMSD (Root Mean Square Deviation), RMSF (Root Mean Square Fluctuation), Rg (Radius of Gyration), SASA (Solvent Accessible Surface Area), and the number of hydrogen bonds (Hbonds) in the solute. One of the most important aspects in an MD simulation is RMSD, which is usually used to determine the mean variation caused by an atom’s displacement from one frame to another in comparison to a reference frame to determine the extent of structural fluctuations and to assess whether the simulation had reached equilibrium [43,44]. The RMSD values obtained in this study, averaging within a range of 0.425 Å, suggest the structure remained stable throughout the simulation compared to the control complex. This reduced level of RMSD of the VacA/ligand 8 complex indicates fewer conformational changes, affirming the stability of the complex over the entire simulation duration (Fig 7A) [45]. Accordingly, the RMSD values of the selected lead molecule (ligand 8) and the control molecule (ranitidine) demonstrated that the ligand 8 molecule holds overall stability during simulation period, as suggested by the ligand dynamics that completely distinguished to each other (Fig 7B). When describing a protein, the RMSF value is crucial since it provides valuable insights into both the local fluctuations within the protein and its overall structural changes along the protein chain [46,47]. The RMSF analysis identified fluctuations in particular residues of the VacA p55 domain when bound to the ligand 8, with distinguishable fluctuations compared to the control. However, initial and final residues found in the VacC A p55 domain were found to be more flexible since they are mostly fluctuating amino acids (Fig 7C). Moreover, ligand 8 itself exhibited minimal fluctuation, indicating lower flexibility.

To reveal the compactness of the complex, the Radius of Gyration (Rg) and solvent-accessible surface area (SASA) were used as counterparts. Consistent low-level fluctuations found in Rg suggesting increased system compactness and rigidity, with a corresponding decrease in SASA. A system’s compactness and rigidity tend to increase when there is a consistent, lower fluctuation rate observed during simulation as shown in Figs 7D and 7E. The average SASA score of the docking complex (VacA p55 domain and ligand 8) was notably below 205 nm² than the control, indicating a significant decrease in the surface area. Thus, Rg and SASA complementarily fitted to each other over the simulation period. Additionally, formation of hydrogen (H) bonds play a crucial role in stabilizing the ligand within the target protein, while also influencing drug selectivity, metabolic rate, and absorption efficiency [48,49]. The number of hydrogen bond formations remained significantly lower, which suggests complex’s favourability during the course of 100 ns MD (Fig 7F). Therefore, the aforementioned analyses suggest the overall stability and flexibility of the docked complex between the p55 domain and the lead compound.

Furthermore, a comprehensive analysis of Principal Components (PCs) of the MD trajectories and measurement of the Gibbs free energy indicated the suitable and firmed leading complex (VacA/ligand 8) compared to the control in the case of dynamics and thermodynamics properties [50]. Most importantly, the overall area captured from dancing motion projected through two-dimensional (2D) spaces for both ligand 8 and VacA/ligand 8 complex was remarkably smaller than the control (Figs 8A and 8B). These findings also signify a favourable and comparatively stable conformation in both cases of dynamics and thermodynamics (Fig 8C–8F). Meanwhile, MM/GBSA analysis of lead-VacA and control-VacA of p55 domain exhibited a distinguished fluctuation in binding free energy profile. Thus, comparatively stable energy distribution observed for the lead compound complex suggests more persistent interactions with the VacA binding pocket relative to the control molecule (Fig 9A–9D). These findings also reciprocally support subsequent computational approaches stated aforementioned including molecular docking, MD trajectories, PCA and FEL. Therefore, the selection of the lead compound (ligand 8) through downstream process was based on multiparameter decision matrix, not a single parameter alone.

In *in-silico* research, there is an extensive interest in developing novel drugs against various bacterial diseases. On the other hand, an emerging and increasingly indispensable area of research involves targeting bacterial virulence factors to mitigate infection and facilitate the development of novel therapeutic agents. Thus, an *in silico* technique to prevent *H. pylori* infection could be used alone or in conjunction with already prescribed medications. Moreover, this strategy has the potential to improve treatment specificity and achieve greater eradication efficacy of the pathogen, *H. pylori*. This study thus adopted a drug design strategy targeting VacA rather than a vaccine-based approach. While vaccine development represents a promising prophylactic strategy against *H. pylori*, several challenges remain, including antigenic variability, limited long-term protective efficacy, and difficulties in generating robust mucosal immune responses. In contrast, targeting VacA with small-molecule inhibitors offers a complementary therapeutic approach capable of directly neutralizing toxin-mediated pathogenic effects during infection [51]. We also propose experimental validation strategies that emphasize the predictive nature of our findings and outline a feasible path toward empirical validation. Specifically, we propose (i) recombinant VacA binding assays to confirm direct ligand–protein interaction, (ii) surface plasmon resonance (SPR) analysis to quantify binding affinity and kinetics, (iii) VacA-induced vacuolation assays in gastric epithelial cells to assess functional inhibition, (iv) oligomerization interference studies to determine effects on toxin assembly, (v) electrophysiological assays to evaluate potential disruption of pore-forming activity, and (vi) cytotoxicity testing to examine compound safety profiles. Together, these approaches provide a structured experimental roadmap to validate the computationally identified *in silico* hit and rigorously assess its biological relevance.

### Study limitations

Although this study is enabling fundamental findings through ground research with a full of computational mechanistic way, this requires robust empirical validation as well as further strategies to be addressed. Similarly, single trajectory simulations were performed; replicate simulations were not conducted due to computational constraints. Therefore, functional interpretations derived from the MD results are the part of hypothesis-driven observation rather than definitive mechanistic conclusions. Although QSAR-based ADMET tools provide valuable early-stage insights, their predictions are inherently dependent on training datasets and model assumptions, which may introduce uncertainty. Additionally, these analyses do not fully capture complex metabolic pathways or enzyme-mediated biotransformation processes that occur *in-vivo*. Therefore, the predicted pharmacokinetic and toxicity profiles should be considered preliminary and require confirmation through experimental ADME and toxicity studies. Additionally, it is crucial to uncover the underlying mechanisms of VacA’s multifunctionality and understand how its coordinated actions contribute to *H. pylori* colonization, persistence in the gastric environment, and evasion of the host immune defences. The single reference molecule was taken to consider as a representative chemical that primarily used in gastric disorder including heartburn, Gastroesophageal Reflux Diseases (GERD), stomach ulcers etc. However, having strong pharmacological relevance of *H. pylori* associated complication, this reference molecule was taken into consideration.

## Conclusion

Vacuolating cytotoxin A (VacA) triggers a variety of cellular effects that contribute to significant and uncontrolled damage to the gastrointestinal tract. To prevent VacA-mediated cellular damage, it is indispensable to develop novel antimicrobials that can inhibit VacA production by *H. pylori*. This study found several potential ligand molecules from ZINC 15 database with excellent pharmacological properties (ADMET) and high binding affinities towards the p55 domain of the VacA toxin produced by *H. pylori*; thus, they are supposed to inhibit VacA attaching to the epithelial plasma membrane of the host cells. Hence, these selected compounds would be capable of preventing chronic gastritis and other diseases by interfering with the formation of anion channels, causing vacuoles, and enabling the infiltration of pathogenic *H. pylori* into epithelial cells. Among the selected ligand compounds, ZINC4004291 ((3As,4R,9bS)-4-pyridin-4-yl-8-(trifluoromethyl)-3a,4,5,9b-tetrahydro-3H-cyclopenta[c]quinolone; PubChem ID: 7070714) was the promising one due to its good binding affinity and low toxicity. Furthermore, MD simulation of the identified compound, ZINC4004291 showed the minimum levels of the RMSD, RMSF, Rg, and the SASA values, suggesting the overall stability of the ligand to the p55 domain of VacA protein. However, substantial *in-vitro* research is required to validate the efficacy of the selected lead, ZINC4004291, in preventing *H. pylori*-mediated infection and blocking the p55 domain of the VacA toxin.

## Supporting information

S1 Fig Crystal structure of the *Helicobacter pylori* vacuolating toxin p55 domain (PDB ID: 2QV3). (DOCX)

S2 Fig Homology modeled VacA by Phyre2 server. (DOCX)

S3 Fig Refined structure by Galaxy Refiner. (DOCX)

S4 Fig Chemical structure of selected ligands. (DOCX)

S5 Fig Interactions of ligands with p55 domain of VacA in 2D and 3D using Discovery Studio. (DOCX)

S6 Fig Interactions of ligands with p55 domain of VacA (Hydrogen Bond) using Discovery Studio. (DOCX)

S1 Table RAST annotation of different strains of *Helicobacter pylori.* (DOCX)

S2 Table List of all ligands that bind to active site and their binding affinity. (DOCX)

S3 Table Potential ligands and their information. (DOCX)

S4 Table ADMET properties of Ligand 1 ((1S,3Ar,6aR)-5-(4-methoxyphenyl)-1 phenylspiro[3a,6a-dihydro-1H-furo[3,4-c]pyrrole-3,2’-indene]-1’,3’,4,6-tetrone). (DOCX)

S5 Table ADMET properties of Ligand 2 (5-Amino-6-[(2, 5-dimethylindol-3-ylidene) methyl]-2-morpholin-4-yl [1,3,4]thiadiazolo[3,2-a]pyrimidin-7-one). (DOCX)

S6 Table ADMET properties of Ligand 3 (5-[(5-Methyl-3-oxo-2-phenyl-1H-pyrazol-4-yl) methylideneamino]-6-morpholin-4-yl-1,3-dihydrobenzimidazol-2-one). (DOCX)

S7 Table ADMET properties of Ligand 4 (Ethyl (4E)-4-[[(5E)-5-[(3-hydroxyphenyl) methylidene]-4-oxo-2-sulfanylidene-1, 3-thiazolidin-3-yl] methylidene]-5-oxo-1-phenylpyrazole-3-carboxylate). (DOCX)

S8 Table ADMET properties of Ligand 5 (2-[(11,12-Dimethyl-10-thia-3,4,6,8-tetrazatricyclo[7.3.0.02,6]dodeca-1(9),2,4,7,11-pentaen-5-yl)sulfanyl]-1-[4-(4-methoxyphenyl)piperazin-1-yl]ethanone). (DOCX)

S9 Table ADMET properties of Ligand 6 (1-Acetyl-3-{3-[2-(3, 4-dimethoxyphenyl) ethyl]-4-oxo-2-thioxo (1, 3-thiazolidin-5-ylidene)}-2-oxobenzo[d]azoline). (DOCX)

S10 Table ADMET properties of Ligand 7 ((3Ar, 4R, 9bS)-4-pyridin-3-yl-8-(trifluoromethyl)-3a, 4, 5,9b-tetrahydro-3H-cyclopenta[c]quinolone). (DOCX)

S11 Table ADMET properties of Ligand 8 ((3As, 4R, 9bS)-4-pyridin-4-yl-8-(trifluoromethyl)-3a, 4, 5,9b-tetrahydro-3H-cyclopenta[c]quinolone). (DOCX)

S12 Table ADMET properties of reference drug molecule, Ranitidine. (DOCX)
