# Supplementary material for: Structure-guided identification of a potential inhibitor targeting the VacA toxin of Helicobacter pylori
Source: PLoS One. 2026 Jul 22;21(7):e0354383. doi: 10.1371/journal.pone.0354383 (PMC13390867; doi:10.1371/journal.pone.0354383)
Supplement: S1 Fig — (DOCX) [file pone.0354383.s001.docx]

**Crystal Structure of the *Helicobacter pylori* Vacuolating Toxin p55 Domain**

>2QV3_1|Chain A|Vacuolating cytotoxin|*Helicobacter pylori*

TVVNIDRINTKADGTIKVGGFKASLTTNAAHLNIGKGGVNLSNQASGRTLLVENLTGNITVDGPLRVNNQVGGYALAGSSANFEFKAGVDTKNGTATFNNDISLGRFVNLKVDAHTANFKGIDTGNGGFNTLDFSGVTNKVNINKLITASTNVAVKNFNINELIVKTNGVSVGEYTHFSEDIGSQSRINTVRLETGTRSIFSGGVKFKSGEKLVIDEFYYSPWNYFDARNIKNVEITRKFASSTPENPWGTSKLMFNNLTLGQNAVMDYSQFSNLTIQGDFINNQGTINYLVRGGKVATLNVGNAAAMMFNNDIDSATGFYKPLIKINSAQDLIKNTEHVLLKAKIIGYGNVSTGTNGISNVNLEEQFKERLALYNNNNRMDTCVVRNTDDIKACGMAIGNQSMVNNPDNYKYLIGKAWKNIGISKTANGSKISVYYLGNSTPTENGGNTTNLPTNT


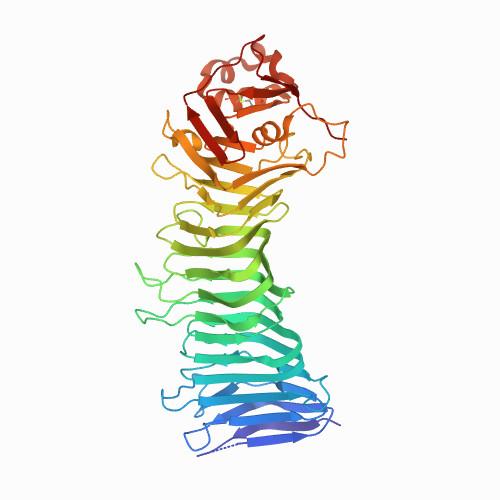


**S1 Fig:** Crystal structure of the *Helicobacter pylori* vacuolating toxin p55 domain (PDB ID: 2QV3).
