# Supplementary figures and images for: Structure-guided identification of a potential inhibitor targeting the VacA toxin of Helicobacter pylori
Source: PLoS One. 2026 Jul 22;21(7):e0354383. doi: 10.1371/journal.pone.0354383 (PMC13390867; doi:10.1371/journal.pone.0354383)

**
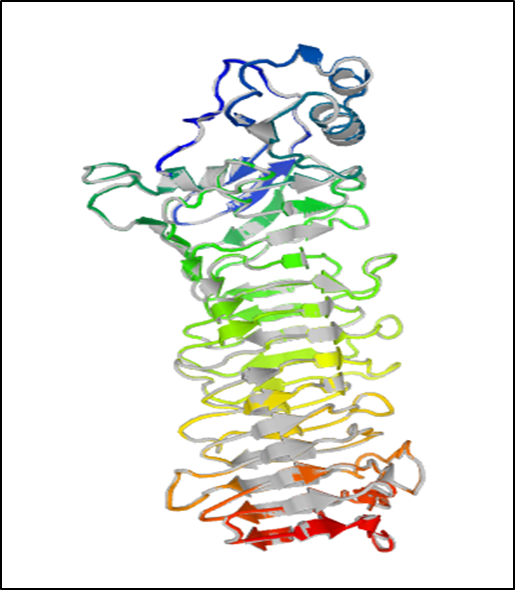
**

**S2 Fig:** Homology modeled VacA by Phyre2 server.

Supplement: S2 Fig — (DOCX) [file pone.0354383.s002.docx]

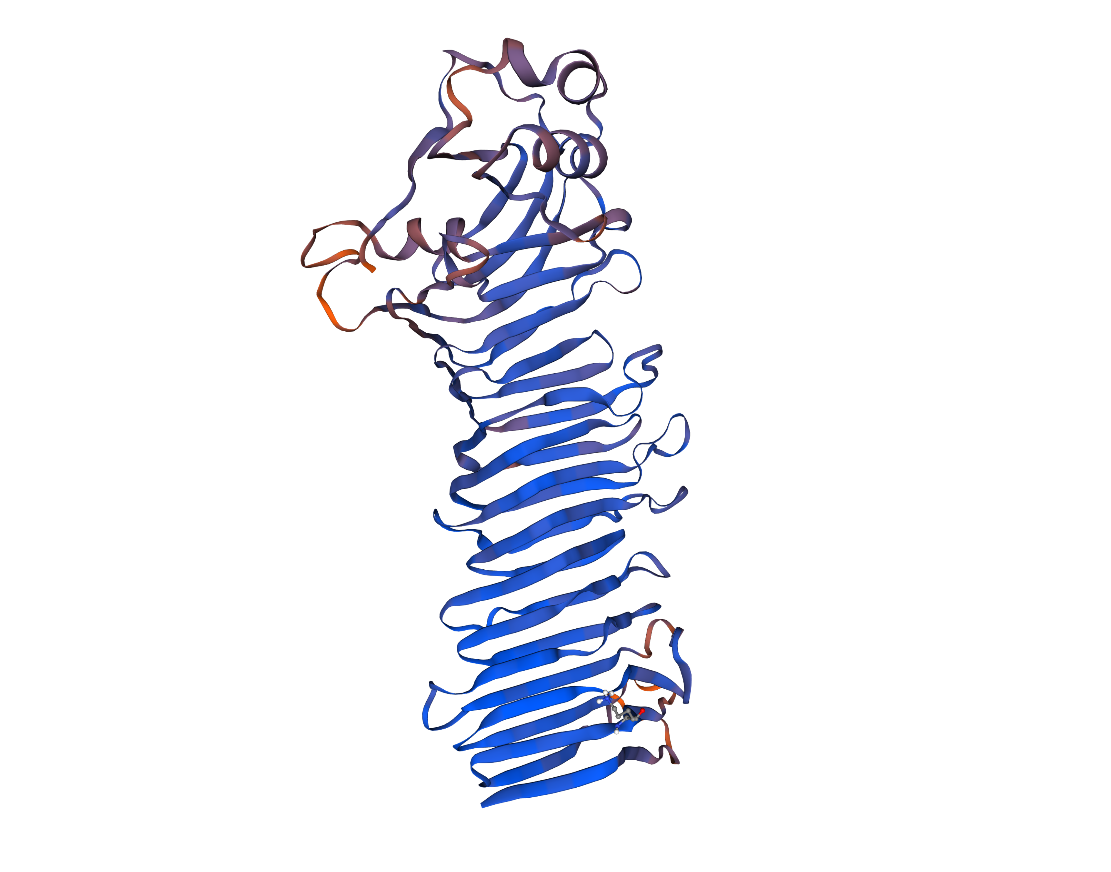


**S3 Fig:** Refined structure by Galaxy Refiner.

Supplement: S3 Fig — (DOCX) [file pone.0354383.s003.docx]
