# Supplementary material for: Structure-guided identification of a potential inhibitor targeting the VacA toxin of Helicobacter pylori
Source: PLoS One. 2026 Jul 22;21(7):e0354383. doi: 10.1371/journal.pone.0354383 (PMC13390867; doi:10.1371/journal.pone.0354383)
Supplement: S4 Fig — (DOCX) [file pone.0354383.s004.docx]

| 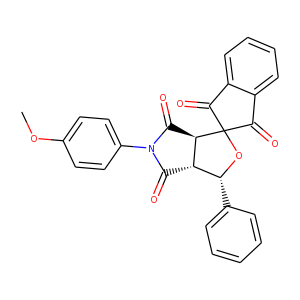 | 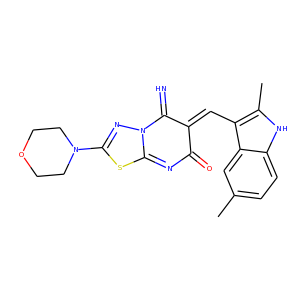 |
| --- | --- |
| **ZINC9086561 (ligand 1)**: (1S,3Ar,6aR)-5-(4-methoxyphenyl)-1-phenylspiro[3a,6a-dihydro-1H-furo[3,4-c]pyrrole-3,2'-indene]-1',3',4,6-tetrone | **ZINC41084323 (ligand 2):** 5-Amino-6-[(2,5-dimethylindol-3-ylidene)methyl]-2-morpholin-4-yl-[1,3,4]thiadiazolo[3,2-a]pyrimidin-7-one |
| 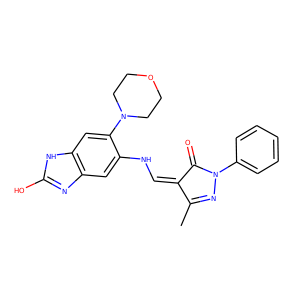 | 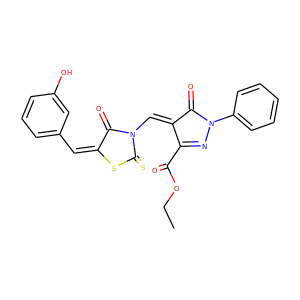 |
| **ZINC408534388 (ligand 3)**: 5-[(5-Methyl-3-oxo-2-phenyl-1H-pyrazol-4-yl)methylideneamino]-6-morpholin-4-yl-1,3-dihydrobenzimidazol-2-one | **ZINC8665141 (ligand 4)**: Ethyl (4E)-4-[[(5E)-5-[(3-hydroxyphenyl)methylidene]-4-oxo-2-sulfanylidene-1,3-thiazolidin-3-yl]methylidene]-5-oxo-1-phenylpyrazole-3-carboxylate |
| 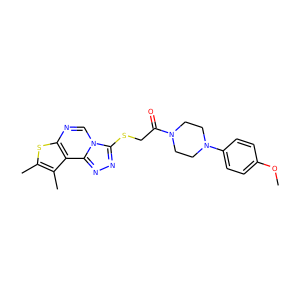 | 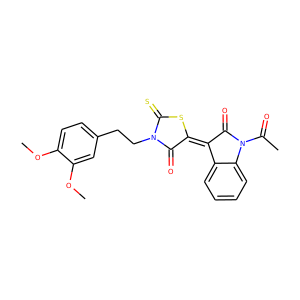 |
| **ZINC1379186 (ligand 5):** 2-[(11,12-Dimethyl-10-thia-3,4,6,8-tetrazatricyclo[7.3.0.02,6]dodeca-1(9),2,4,7,11-pentaen-5-yl)sulfanyl]-1-[4-(4-methoxyphenyl)piperazin-1-yl]ethanone | **ZINC8821620 (ligand 6)**: 1-Acetyl-3-{3-[2-(3,4-dimethoxyphenyl)ethyl]-4-oxo-2-thioxo(1,3-thiazolidin-5-ylidene)}-2-oxobenzo[d]azoline |
| 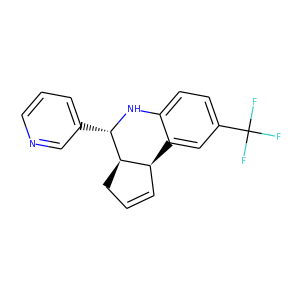 | 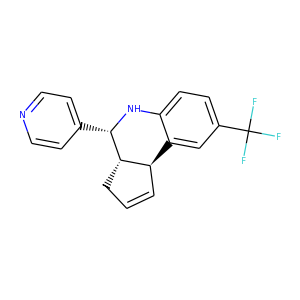 |
| **ZINC3644748 (ligand 7)**: (3Ar,4R,9bS)-4-pyridin-3-yl-8-(trifluoromethyl)-3a,4,5,9b-tetrahydro-3H-cyclopenta[c]quinolone | **ZINC4004291 (ligand 8)**: (3As,4R,9bS)-4-pyridin-4-yl-8-(trifluoromethyl)-3a,4,5,9b-tetrahydro-3H-cyclopenta[c]quinolone |

**S4 Fig:** Chemical structure of selected ligands.
