# Supplementary material for: Structure-guided identification of a potential inhibitor targeting the VacA toxin of Helicobacter pylori
Source: PLoS One. 2026 Jul 22;21(7):e0354383. doi: 10.1371/journal.pone.0354383 (PMC13390867; doi:10.1371/journal.pone.0354383)
Supplement: S1 Table — (DOCX) [file pone.0354383.s007.docx]

**S1 Table:** RAST annotation of different strains of *Helicobacter pylori.*

| **SN** | **Strain** | **Category** | **Subcategory** | **Subsystem** | **Role** |
| --- | --- | --- | --- | --- | --- |
| 1 | *Helicobacter pylori* puno135 | Virulence, Disease and Defense | Toxins and super antigens | Helicobacter vacuolating cytotoxin | vacuolating cytotoxin |
| 2 | *Helicobacter pylori* strain ATCC43504 | Virulence, Disease and Defense | Toxins and super antigens | Helicobacter vacuolating cytotoxin | vacuolating cytotoxin |
| 3 | *Helicobacter pylori* PMSS1 | Virulence, Disease and Defense | Toxins and super antigens | Helicobacter vacuolating cytotoxin | vacuolating cytotoxin |
| 4 | *Helicobacter pylori* Hp A-11 | Virulence, Disease and Defense | Toxins and super antigens | Helicobacter vacuolating cytotoxin | vacuolating cytotoxin |
| 5 | *Helicobacter pylori* FDAARGOS_299 | Virulence, Disease and Defense | Toxins and super antigens | Helicobacter vacuolating cytotoxin | vacuolating cytotoxin |
