# Supplementary material for: Structure-guided identification of a potential inhibitor targeting the VacA toxin of Helicobacter pylori
Source: PLoS One. 2026 Jul 22;21(7):e0354383. doi: 10.1371/journal.pone.0354383 (PMC13390867; doi:10.1371/journal.pone.0354383)
Supplement: S2 Table — (DOCX) [file pone.0354383.s008.docx]

**S2 Table:** List of all ligands that bind to active site and their binding affinity.

| **SL** | **Ligand ID (ZINC15)** | **Binding Energy** |  | **SL** | **Ligand ID (ZINC15)** | **Binding Energy** |
| --- | --- | --- | --- | --- | --- | --- |
| 1 | ZINC00009086561 | -8.2 |  | 45 | ZINC000012521833 | -6.7 |
| 2 | ZINC000041084323 | -7.8 |  | 46 | ZINC000013675519 | -6.7 |
| 3 | ZINC000408534388 | -7.8 |  | 47 | ZINC000019714243 | -6.7 |
| 4 | ZINC000008665141 | -7.3 |  | 48 | ZINC000020481468 | -6.7 |
| 5 | ZINC000001379186 | -7.3 |  | 49 | ZINC000022448618 | -6.7 |
| 6 | ZINC000008821620 | -7.2 |  | 50 | ZINC000090485761 | -6.7 |
| 7 | ZINC000003644748 | -7 |  | 51 | ZINC000102738494 | -6.7 |
| 8 | ZINC000004004291 | -6.9 |  | 52 | ZINC000107745729 | -6.7 |
| 9 | ZINC000072321994 | -6.9 |  | 53 | ZINC000214685209 | -6.7 |
| 10 | ZINC000101123620 | -6.9 |  | 54 | ZINC000222700449 | -6.7 |
| 11 | ZINC000101123681 | -6.9 |  | 55 | ZINC000223879279 | -6.7 |
| 12 | ZINC000101842344 | -6.9 |  | 56 | ZINC000223941809 | -6.7 |
| 13 | ZINC000101842353 | -6.9 |  | 57 | ZINC000224515481 | -6.7 |
| 14 | ZINC000116034066 | -6.9 |  | 58 | ZINC000225539479 | -6.7 |
| 15 | ZINC000223708539 | -6.9 |  | 59 | ZINC000227173438 | -6.7 |
| 16 | ZINC000223849748 | -6.9 |  | 60 | ZINC000408769463 | -6.7 |
| 17 | ZINC000225527718 | -6.9 |  | 61 | ZINC000408776993 | -6.7 |
| 18 | ZINC000225527718 | -6.9 |  | 62 | ZINC000409213696 | -6.7 |
| 19 | ZINC000225527718 | -6.9 |  | 63 | ZINC000000624266 | -6.6 |
| 20 | ZINC000225539452 | -6.9 |  | 64 | ZINC000001353708 | -6.6 |
| 21 | ZINC000408765396 | -6.9 |  | 65 | ZINC000004008026 | -6.6 |
| 22 | ZINC000409213191 | -6.9 |  | 66 | ZINC000004008026 | -6.6 |
| 23 | ZINC000409215108 | -6.9 |  | 67 | ZINC000013648340 | -6.6 |
| 24 | ZINC000409218793 | -6.9 |  | 68 | ZINC000013649317 | -6.6 |
| 25 | ZINC000004086060 | -6.8 |  | 69 | ZINC000020249070 | -6.6 |
| 26 | ZINC000004086060 | -6.8 |  | 70 | ZINC000033666318 | -6.6 |
| 27 | ZINC000004622747 | -6.8 |  | 71 | ZINC000035226009 | -6.6 |
| 28 | ZINC000016951202 | -6.8 |  | 72 | ZINC000102882473 | -6.6 |
| 29 | ZINC000019659855 | -6.8 |  | 73 | ZINC000103918229 | -6.6 |
| 30 | ZINC000020265604 | -6.8 |  | 74 | ZINC000108074751 | -6.6 |
| 31 | ZINC000020459596 | -6.8 |  | 75 | ZINC000108997358 | -6.6 |
| 32 | ZINC000033762026 | -6.8 |  | 76 | ZINC000408548612 | -6.6 |
| 33 | ZINC000101488159 | -6.8 |  | 77 | ZINC000408783265 | -6.6 |
| 34 | ZINC000103461748 | -6.8 |  | 78 | ZINC000409212205 | -6.6 |
| 35 | ZINC000219011023 | -6.8 |  | 79 | ZINC000409214201 | -6.6 |
| 36 | ZINC000225527671 | -6.8 |  | 80 | ZINC000001236989 | -6.5 |
| 37 | ZINC000225527750 | -6.8 |  | 81 | ZINC000001300562 | -6.5 |
| 38 | ZINC000409201413 | -6.8 |  | 82 | ZINC000001361479 | -6.5 |
| 39 | ZINC000409202677 | -6.8 |  | 83 | ZINC000004140490 | -6.5 |
| 40 | ZINC000409202678 | -6.8 |  | 84 | ZINC000006571320 | -6.5 |
| 41 | ZINC000409214205 | -6.8 |  | 85 | ZINC000006714568 | -6.5 |
| 42 | ZINC000004914394 | -6.7 |  | 86 | ZINC000013668449 | -6.5 |
| 43 | ZINC000008819872 | -6.7 |  | 87 | ZINC000013675466 | -6.5 |
| 44 | ZINC000009232377 | -6.7 |  | 88 | ZINC000033542666 | -6.5 |
| 89 | ZINC000102964710 | -6.5 |  | 134 | ZINC000017141720 | -6.1 |
| 90 | ZINC000107701940 | -6.5 |  | 135 | ZINC000020226865 | -6.1 |
| 91 | ZINC000219191152 | -6.5 |  | 136 | ZINC000033540077 | -6.1 |
| 92 | ZINC000223850714 | -6.5 |  | 137 | ZINC000090485762 | -6.1 |
| 93 | ZINC000223889591 | -6.5 |  | 138 | ZINC000225115865 | -6.1 |
| 94 | ZINC000408601776 | -6.5 |  | 139 | ZINC000408772660 | -6.1 |
| 95 | ZINC000408601777 | -6.5 |  | 140 | ZINC000408778655 | -6.1 |
| 96 | ZINC000408777773 | -6.5 |  | 141 | ZINC000001301649 | -6 |
| 97 | ZINC000001590579 | -6.4 |  | 142 | ZINC000006544969 | -6 |
| 98 | ZINC000004914375 | -6.4 |  | 143 | ZINC000020356356 | -6 |
| 99 | ZINC000016024874 | -6.4 |  | 144 | ZINC000033539710 | -6 |
| 100 | ZINC000016756737 | -6.4 |  | 145 | ZINC000033540076 | -6 |
| 101 | ZINC000020226863 | -6.4 |  | 146 | ZINC000033544266 | -6 |
| 102 | ZINC000033543851 | -6.4 |  | 147 | ZINC000033589735 | -6 |
| 103 | ZINC000033543852 | -6.4 |  | 148 | ZINC000054361152 | -6 |
| 104 | ZINC000035167917 | -6.4 |  | 149 | ZINC000408785294 | -6 |
| 105 | ZINC000225527698 | -6.4 |  | 150 | ZINC000017130347 | -5.9 |
| 106 | ZINC000225527698 | -6.4 |  | 151 | ZINC000033538764 | -5.9 |
| 107 | ZINC000225527698 | -6.4 |  | 152 | ZINC000033542791 | -5.9 |
| 108 | ZINC000004093428 | -6.3 |  | 153 | ZINC000054361201 | -5.9 |
| 109 | ZINC000011859247 | -6.3 |  | 154 | ZINC000101842208 | -5.9 |
| 110 | ZINC000016362544 | -6.3 |  | 155 | ZINC000104292208 | -5.9 |
| 111 | ZINC000017023279 | -6.3 |  | 156 | ZINC000104389819 | -5.9 |
| 112 | ZINC000033542295 | -6.3 |  | 157 | ZINC000224954048 | -5.9 |
| 113 | ZINC000033542790 | -6.3 |  | 158 | ZINC000409217203 | -5.9 |
| 114 | ZINC000044903572 | -6.3 |  | 159 | ZINC000004093427 | -5.8 |
| 115 | ZINC000100931036 | -6.3 |  | 160 | ZINC000004933335 | -5.8 |
| 116 | ZINC000104304738 | -6.3 |  | 161 | ZINC000006907192 | -5.8 |
| 117 | ZINC000225539407 | -6.3 |  | 162 | ZINC000008104605 | -5.8 |
| 118 | ZINC000225539407 | -6.3 |  | 163 | ZINC000225139160 | -5.8 |
| 119 | ZINC000225539407 | -6.3 |  | 164 | ZINC000225400938 | -5.8 |
| 120 | ZINC000408773199 | -6.3 |  | 165 | ZINC000004745552 | -5.7 |
| 121 | ZINC000001240495 | -6.2 |  | 166 | ZINC000004758385 | -5.7 |
| 122 | ZINC000001245921 | -6.2 |  | 167 | ZINC000033538604 | -5.7 |
| 123 | ZINC000001251286 | -6.2 |  | 168 | ZINC000033544756 | -5.7 |
| 124 | ZINC000004933237 | -6.2 |  | 169 | ZINC000104304764 | -5.7 |
| 125 | ZINC000005609975 | -6.2 |  | 170 | ZINC000224720193 | -5.7 |
| 126 | ZINC000005977584 | -6.2 |  | 171 | ZINC000409218596 | -5.7 |
| 127 | ZINC000006907162 | -6.2 |  | 172 | ZINC000008104689 | -5.6 |
| 128 | ZINC000020140258 | -6.2 |  | 173 | ZINC000033539737 | -5.6 |
| 129 | ZINC000020356358 | -6.2 |  | 174 | ZINC000033539738 | -5.6 |
| 130 | ZINC000033539711 | -6.2 |  | 175 | ZINC000033544267 | -5.6 |
| 131 | ZINC000104292203 | -6.2 |  | 176 | ZINC000104389824 | -5.6 |
| 132 | ZINC000409201399 | -6.2 |  | 177 | ZINC000409213350 | -5.6 |
| 133 | ZINC000005609961 | -6.1 |  | 178 | ZINC000033545378 | -5.5 |
