# Supplementary material for: Structure-guided identification of a potential inhibitor targeting the VacA toxin of Helicobacter pylori
Source: PLoS One. 2026 Jul 22;21(7):e0354383. doi: 10.1371/journal.pone.0354383 (PMC13390867; doi:10.1371/journal.pone.0354383)
Supplement: S3 Table — (DOCX) [file pone.0354383.s009.docx]

**S3 Table:** Potential ligands and their information.

| **SL** | **ZINC15 ID** | **PubChem CID** | **Compound Name** | **Molecular Formula** | **Molecular Weight**  (g/mol) | **H bond Donor** | **H Bond Acceptor** | **LogP** | **Molar Refractivity** |
| --- | --- | --- | --- | --- | --- | --- | --- | --- | --- |
|  | ZINC9086561 | 40928476 | (1S,3Ar,6aR)-5-(4-methoxyphenyl)-1-phenylspiro[3a,6a-dihydro-1H-furo[3,4-c]pyrrole-3,2'-indene]-1',3',4,6-tetrone | C_27_H_19_NO_6_ | 453.4 | 0 | 7 | 3.157599 | 119.635956 |
|  | ZINC41084323 | 52098094 | 5-Amino-6-[(2,5-dimethylindol-3-ylidene)methyl]-2-morpholin-4-yl-[1,3,4]thiadiazolo[3,2-a]pyrimidin-7-one | C_20_H_20_N6O_2_S | 408.5 | 2 | 7 | 2.154230 | 113.989388 |
|  | ZINC408534388 | 137070061 | 5-[(5-Methyl-3-oxo-2-phenyl-1H-pyrazol-4-yl)methylideneamino]-6-morpholin-4-yl-1,3-dihydrobenzimidazol-2-one | C_22_H_22_N_6_O_3_ | 418.4 | 3 | 9 | 3.008299 | 120.031075 |
|  | ZINC8665141 | 92341149 | Ethyl (4E)-4-[[(5E)-5-[(3-hydroxyphenyl)methylidene]-4-oxo-2-sulfanylidene-1,3-thiazolidin-3-yl]methylidene]-5-oxo-1-phenylpyrazole-3-carboxylate | C_23_H_17_N_3_O_5_S_2_ | 479.5 | 1 | 8 | 3.443199 | 129.197769 |
|  | ZINC1379186 | 1468378 | 2-[(11,12-Dimethyl-10-thia-3,4,6,8-tetrazatricyclo[7.3.0.02,6]dodeca-1(9),2,4,7,11-pentaen-5-yl)sulfanyl]-1-[4-(4-methoxyphenyl)piperazin-1-yl]ethanone | C_22_H_24_N_6_O_2_S_2_ | 468.6 | 0 | 7 | 3.405339 | 128.149994 |
|  | ZINC8821620 | 6137085 | 1-Acetyl-3-{3-[2-(3,4-dimethoxyphenyl)ethyl]-4-oxo-2-thioxo(1,3-thiazolidin-5-ylidene)}-2-oxobenzo[d]azoline | C_23_H_20_N_2_O_5_S_2_ | 468.5 | 0 | 7 | 3.410999 | 126.680969 |
|  | ZINC3644748 | 11865361 | (3Ar,4R,9bS)-4-pyridin-3-yl-8-(trifluoromethyl)-3a,4,5,9b-tetrahydro-3H-cyclopenta[c]quinoline | C_18_H_15_F_3_N_2_ | 316.3 | 1 | 2 | 4.926900 | 82.032684 |
|  | ZINC4004291 | 7070714 | (3As,4R,9bS)-4-pyridin-4-yl-8-(trifluoromethyl)-3a,4,5,9b-tetrahydro-3H-cyclopenta[c]quinoline | C_18_H_15_F_3_N_2_ | 316.3 | 1 | 2 | 4.926900 | 82.032684 |
