# Supplementary material for: Structure-guided identification of a potential inhibitor targeting the VacA toxin of Helicobacter pylori
Source: PLoS One. 2026 Jul 22;21(7):e0354383. doi: 10.1371/journal.pone.0354383 (PMC13390867; doi:10.1371/journal.pone.0354383)
Supplement: S4 Table — (DOCX) [file pone.0354383.s010.docx]

**S4 Table:** ADMET properties of Ligand 1 ((1S,3Ar,6aR)-5-(4-methoxyphenyl)-1 phenylspiro[3a,6a-dihydro-1H-furo[3,4-c]pyrrole-3,2'-indene]-1',3',4,6-tetrone).

| **Property** | **Model Name** | **Predicted Value** | **Unit** |
| --- | --- | --- | --- |
| **Absorption** | Water solubility | **-4.69** | Numeric (log mol/L) |
|  | Caco2 permeability | **1.3** | Numeric (log Papp in 10 cm/s) |
|  | Intestinal absorption (human) | **100** | Numeric (% Absorbed) |
|  | Skin Permeability | **-2.735** | Numeric (log Kp) |
|  | P-glycoprotein substrate | **No** | Categorical (Yes/No) |
|  | P-glycoprotein I inhibitor | **Yes** | Categorical (Yes/No) |
|  | P-glycoprotein II inhibitor | **Yes** | Categorical (Yes/No) |
| **Distribution** | VDss (human) | **-1.141** | Numeric (log L/kg) |
|  | Fraction unbound (human) | **0.053** | Numeric (Fu) |
|  | BBB permeability | **-0.707** | Numeric (log BB) |
|  | CNS permeability | **-2.969** | Numeric (log PS) |
| **Metabolism** | CYP2D6 substrate | **No** | Categorical (Yes/No) |
|  | CYP3A4 substrate | **Yes** | Categorical (Yes/No) |
|  | CYP1A2 inhibitor | **No** | Categorical (Yes/No) |
|  | CYP2C19 inhibitor | **Yes** | Categorical (Yes/No) |
|  | CYP2C9 inhibitor | **Yes** | Categorical (Yes/No) |
|  | CYP2D6 inhibitor | **No** | Categorical (Yes/No) |
|  | CYP3A4 inhibitor | **Yes** | Categorical (Yes/No) |
| **Excretion** | Total Clearance | **-0.094** | Numeric (log ml/min/kg) |
|  | Renal OCT2 substrate | **No** | Categorical (Yes/No) |
| **Toxicity** | AMES toxicity | **Yes** | Categorical (Yes/No) |
|  | Max. tolerated dose (human) | **0.631** | Numeric (log mg/kg/day) |
|  | hERG I inhibitor | **No** | Categorical (Yes/No) |
|  | hERG II inhibitor | **Yes** | Categorical (Yes/No) |
|  | Oral Rat Acute Toxicity (LD50) | **2.669** | Numeric (mol/kg) |
|  | Oral Rat Chronic Toxicity (LOAEL) | **1.706** | Numeric (log mg/kg_bw/day) |
|  | Hepatotoxicity | **Yes** | Categorical (Yes/No) |
|  | Skin Sensitisation | **No** | Categorical (Yes/No) |
|  | *T. Pyriformis* toxicity | **0.285** | Numeric (log ug/L) |
|  | Minnow toxicity | **-2.39** | Numeric (log mM) |
