# Supplementary material for: Structure-guided identification of a potential inhibitor targeting the VacA toxin of Helicobacter pylori
Source: PLoS One. 2026 Jul 22;21(7):e0354383. doi: 10.1371/journal.pone.0354383 (PMC13390867; doi:10.1371/journal.pone.0354383)
Supplement: S12 Table — (DOCX) [file pone.0354383.s018.docx]

**Review report**

The study addresses an important problem—anti-virulence targeting of VacA as an alternative to antibiotics, and applies a standard CADD pipeline (homology modeling → docking → ADMET → MD simulation → PCA/FEL). The workflow is coherent and technically competent. However, several issues need strengthening, especially methodological justification and reproducibility.

**1. Novelty and scope:** Targeting VacA p55 domain via in silico screening has been reported previously. Then, what’s the novelty of this work?

2. Control molecule choice Ranitidine is used as a reference compound, but it is not a VacA inhibitor and does not mechanistically target VacA. Then why did you choose ranitidine as a control molecule?

3. Docking protocols and reproducibility

Some essential details need to mention clearly. For example,

- Grid box center coordinates and dimensions
- Exhaustiveness value used in AutoDock Vina
- Whether protein flexibility was considered
- Docking validation strategy (re-docking, RMSD threshold)

4. Binding affinity interpretation

The docking score of ligand 8 is –6.9 kcal/mol, which is moderate, and not so strong. Several other ligands show higher affinity (e.g., –8.2, –7.8 kcal/mol) than ligand 8. Then why did you choose ligand 8 as potential therapeutics?

5. MD simulation rigor

Although MD simulation was performed rigorously with various parameters (e.g. 100 ns MD, RMSD, RMSF, Rg, SASA, H-bonds, PCA, FEL), there remains some concerns such as no replicate simulations were performed and no MM/PBSA or MM/GBSA binding free energy was analyzed.

6. Overstatement of biological implications

In the introduction, the statement implying “proposed inhibitors could prevent gastritis and peptic ulcers by preventing the *H. pylori* attachment, colonization, and survival in the host cells” is not experimentally validated and seems overstated. Replace “could prevent gastritis and peptic ulcers by preventing the *H. pylori* attachment, colonization, and survival in the host cells” with “may interfere with VacA-mediated host interactions and the survival of *H. pylori*, although the experimental validation remains pending.”

**7. Title:** “Structure-guided discovery” is suitable, but slightly overstated. Replace the title with “Structure-guided identification of potential inhibitors targeting the VacA toxin of *Helicobacter pylori*”.

**8. Other Suggestions:** In Abstract, **r**eplace “has implicated the favorable level” with “indicated favorable levels”.

- Replace “optimum thermodynamic behavior” with “favorable thermodynamic stability”.
- Use “molecular dynamics simulation” **consistently** instead of “molecular dynamic simulation”.
- Replace “Newzealand” with “New Zealand”
- Replace “Nizeria” with “Nigeria”
