# Supplementary material for: Structure-guided identification of a potential inhibitor targeting the VacA toxin of Helicobacter pylori
Source: PLoS One. 2026 Jul 22;21(7):e0354383. doi: 10.1371/journal.pone.0354383 (PMC13390867; doi:10.1371/journal.pone.0354383)
Supplement: Attachment — Submitted filename: 17042026-Response_to_Reviewers.docx [file pone.0354383.s021.docx]

Dear Editor

We are grateful for your efforts in editing our manuscript and excellent comments and suggestions for us to revise and resubmit our manuscript. We have read carefully reviewers’ all comments and tried our best to revise our manuscript considering all comments and suggestions. Details revision are given below.

Editorial Comments

1. Please ensure that your manuscript meets PLOS ONE's style requirements, including those for file naming. The PLOS ONE style templates can be found at <https://journals.plos.org/plosone/s/file?id=wjVg/PLOSOne_formatting_sample_main_body.pdf> and<https://journals.plos.org/plosone/s/file?id=ba62/PLOSOne_formatting_sample_title_authors_affiliations.pdf>

- We sincerely thank the editor for the suggestion. We went through the manuscript and have carefully revised it according to the requirements of PLOS ONE’s style.

2. Please note that PLOS One has specific guidelines on code sharing for submissions in which author-generated code underpins the findings in the manuscript. In these cases, we expect all author-generated code to be made available without restrictions upon publication of the work. Please review our guidelines at <https://journals.plos.org/plosone/s/materials-and-software-sharing#loc-sharing-code> and ensure that your code is shared in a way that follows best practice and facilitates reproducibility and reuse.

- We did not generate any code to get access to the information of our manuscript. All information and data of our manuscript are available in the manuscript and supplementary information.

3. Thank you for stating the following in the Acknowledgments Section of your manuscript: “This work was partially supported by Noakhali Science and Technology University ResearchCell Grant, 2022–23 (Grant No.: NSTU-RC-BGE-T-23-35).” We note that you have provided additional information within the Acknowledgements Section that is not currently declared in your Funding Statement. Please note that funding information should not appear in the Acknowledgments section or other areas of your manuscript. We will only publish funding information present in the Funding Statement section of the online submission form. Please remove any funding-related text from the manuscript and let us know how you would like to update your Funding Statement. Currently, your Funding Statement reads as follows: “The author(s) received no specific funding for this work.” Please include your amended statements within your cover letter; we will change the online submission form on your behalf.

- We are thankful to the editor for the suggestion. According to the suggestion, we have removed the funding related statement “This work was partially supported by Noakhali Science and Technology University Research Cell Grant, 2022–23 (Grant No.: NSTU-RC-BGE-T-23-35)” from the Acknowledgements Section in our revised manuscript. The Funding Statement will be “The author(s) received no specific funding for this work.” Actually, we didn’t receive any specific funding for this research. However, we used a little money from the said grant to repair the computer facility of Bioinformatics and Computational Biology Laboratory of the Department of Biotechnology and Genetic Engineering, Noakhali Science and Technology University”. Thus, we had mentioned this in the Acknowledgements Section of the manuscript and we have already removed this information from the revised manuscript according to the given suggestion.

4. Thank you for stating the following financial disclosure: “This work was partially supported by Noakhali Science and Technology University ResearchCell Grant, 2022–23 (Grant No.: NSTU-RC-BGE-T-23-35).” Please state what role the funders took in the study.  If the funders had no role, please state: "The funders had no role in study design, data collection and analysis, decision to publish, or preparation of the manuscript." If this statement is not correct you must amend it as needed. Please include this amended Role of Funder statement in your cover letter; we will change the online submission form on your behalf.

- Thank you very much for the suggestion. "The funders had no role in study design, data collection and analysis, decision to publish, or preparation of the manuscript."

5. We note that the grant information you provided in the ‘Funding Information’ and ‘Financial Disclosure’ sections do not match. When you resubmit, please ensure that you provide the correct grant numbers for the awards you received for your study in the ‘Funding Information’ section.

- The authors would like to pay their sincere gratitude to the editor. Actually, we didn’t receive any specific funding for this work. Hence, we have removed the funding information in our revised manuscript.

6. Thank you for stating the following financial disclosure: “The author(s) received no specific funding for this work.” At this time, please address the following queries:

a)  Please clarify the sources of funding (financial or material support) for your study. List the grants or organizations that supported your study, including funding received from your institution.

► This work was partially supported by Noakhali Science and Technology University Research Cell Grant, 2022–23 (Grant No.: NSTU-RC-BGE-T-23-35). There was no specific funding for this study. However, a small amount of money from the said grant was utilized to repair the computer facilities of the Bioinformatics and Computational Biology Laboratory (of the Department of Biotechnology and Genetic Engineering, Noakhali Science and Technology University) used for this study.

b) State what role the funders took in the study. If the funders had no role in your study, please state: “The funders had no role in study design, data collection and analysis, decision to publish, or preparation of the manuscript.”

► The funders had no role in study design, data collection and analysis, decision to publish, or preparation of the manuscript.

c) If any authors received a salary from any of your funders, please state which authors and which funders.

► No author received salary from funders for this study.

d) If you did not receive any funding for this study, please state: “The authors received no specific funding for this work.”

► The authors received no specific funding for this work.

Please include your amended statements within your cover letter; we will change the online submission form on your behalf.

► The authors would like to pay their sincere thanks to the editor for the recommendation and suggestion. We have mentioned the role of the funders and the funding statement in the cover letter as suggested

7. If the reviewer comments include a recommendation to cite specific previously published works, please review and evaluate these publications to determine whether they are relevant and should be cited. There is no requirement to cite these works unless the editor has indicated otherwise.

- Thank you very much for the suggestion. We addressed all the comments from the reviewers carefully. Thus, we have made changes in the revised manuscript and used a few new references. We have also carefully evaluated the suggested previously published works and cited wherever it is necessary.

**Revision of manuscript**

**Comments to the Author:**

**Reviewer #1:**

This work is timely and methodologically ambitious, presenting a coherent computational workflow that targets a virulence factor (VacA p55 domain) with potential translational relevance. However, in its current form the manuscript overstates translational readiness, provides limited methodological transparency, and lacks a sufficiently rigorous lead selection justification and experimental validation plan. A major revision is required to clarify scope, strengthen mechanistic framing, and ensure reproducibility before reconsideration for publication.

- We sincerely thank the reviewer for the thoughtful, detailed, and constructive critique. The comments have helped to significantly improved the scientific rigor, clarity, and scope of our manuscript. We have carefully revised the manuscript to address all concerns, including reframing translational claims, strengthening methodological transparency, clarifying mechanistic rationale, and expanding reproducibility details.

**Major revisions**

Reviewer comment 1: Reframe ZINC4004291 as an in silico hit rather than a drug candidate. Remove language implying immediate translational potential and clearly state that findings are predictive. Provide an experimental validation plan.

- We do fully agree. In the revised manuscript, ZINC4004291 is consistently described as computationally identified in silico hit. In the Abstract section of our revised manuscript, we mentioned “Thus, the ligand ZINC4004291 is a computationally identified in silico hit for a possible drug to treat *H. pylori* infection, while additional experimental validation required” (Page 2, Line 42-44). Moreover, all statements implying translational readiness or therapeutic efficacy have been removed or softened. Additionally, a new **Scope Statement** has been added at the end of the Introduction as this study presents a computational lead discovery framework targeting the VacA p55 domain. The findings are predictive in nature and require biochemical, cellular, and in vivo validation (Line 152-154). A few sentences describing the “Proposed Experimental Validation Strategy” has also been added at the end of the Discussion section as per your suggestion (Page 28, Line 742-751).

Reviewer comment 2: Target rationale and mechanism-

Strengthen the biological rationale for targeting the p55 pocket. Clarify whether the pocket overlaps known functional residues involved in VacA membrane interaction, oligomerization, or pore formation, or if it represents a novel allosteric site. If possible, map the pocket onto established VacA functional interfaces and discuss potential mechanistic implications of ligand binding, with precise citations to the latest structural and functional VacA literature.

- Thank you for your valuable suggestions. We have substantially strengthened this section.

The CASTp-identified pocket was mapped onto known p55 structural interfaces based on PDB: 2QV3 and recent structural literature. Moreover, additional allosteric sites along with pocket were newly investigated by PrankWeb tools (Table 2 and Line 356-363). This step clarified regarding known functional residues corresponding to their position. Hence, the revised Discussion now clarifies the following mechanistic implications of ligand binding (Line 632-641).

- The p55 domain mediates receptor interaction and oligomerization.
- The identified pocket overlaps residues implicated in membrane interaction and structural stabilization.
- The pocket may represent a surface-exposed functional interface rather than a catalytic site.

Hence, now we cautiously stated: “Ligand occupancy within this pocket may interfere with VacA structural assembly or host receptor engagement, although this hypothesis requires experimental validation” in the discussion section (Line 640-641). Furthermore, recent structural and cryo-EM studies have been cited to contextualize the mechanistic hypothesis.

Reviewer comment 3: Methods transparency and reproducibility-

Add a comprehensive methods section with enough detail to enable replication:
• Docking: specify software/version, scoring function, grid center and dimensions, exhaustiveness, number of poses retained per ligand, receptor/ligand preparation steps, and how flexibility was treated (rigid receptor vs. induced-fit considerations).

• MD: specify force field, water model, simulation box type and size, ion concentration, time steps, thermostat settings, equilibration length, production run length, convergence criteria; report whether multiple independent runs were performed.

• Data and scripts: provide supplementary files with docking parameters, input files, and analysis scripts; include a brief description of reproducibility checks (e.g., replicate runs, sensitivity analyses).

• Include a comprehensive table for the top ligands (at least the eight initially highlighted) with docking scores (mean and range), key interactions, and basic physicochemical properties. Explain decisions to deprioritize ligands with similar or better docking scores but unfavorable ADMET, stability, or synthetic feasibility.

- Thank you for your insightful observations. We have significantly expanded the method section with enough details ensuring reproducibility. We have added the following information to the revised manuscript:

Docking Details (Line 216-221):

- Software: AutoDock Vina (version specified)
- Grid center coordinates (X, Y, Z values provided)
- Grid dimensions (Å specified)
- Exhaustiveness: 8
- Number of poses retained per ligand: 9
- Receptor preparation: removal of water, addition of hydrogens, Gasteiger charges
- Ligand preparation: energy minimization via Open Babel
- Flexibility: rigid receptor docking
- Docking validation: redocking RMSD < 2.0 Å

MD Simulation Details:

- GROMACS version specified
- Force field: CHARMM27
- Water model: TIP3P
- Box type: cubic
- Box distance: 1 nm
- Ion concentration: 0.15 M NaCl
- Time step: 2 fs
- NVT equilibration: 100 ps
- NPT equilibration: 100 ps
- Production run: 100 ns
- Thermostat: V-rescale
- Barostat: Parrinello–Rahman (Line 252-253)

However, Single trajectory simulations were performed; replicate simulations were not conducted due to computational constraints. We stated this under the limitation section.

Moreover, Docking parameter files, Coordinate files, MD input files including .xtc, .cpt and .mdp along with input files of the MM/GBSA calculation are available upon request to ensure public access and reproducibility.

Furthermore, we have comprehensively modified Table 2 (Table 3 in our revised manuscript) for the top ligands with docking scores, key interactions, and basic physicochemical properties.

Reviewer comment 4: Lead selection and comparative data-

- Present a side-by-side comparison of all top candidates using objective criteria:
- Docking metrics (scores, pose stability)
- MD stability indicators (average RMSD/RMSF in the binding region, convergence behaviour across replicas if performed)
- ADMET predictions with explicit thresholds and caveats
- Practical considerations (synthetic accessibility, commercial availability)
- Provide a transparent justification for selecting ZINC4004291 as the lead, ensuring the rationale is not solely based on a single docking score.
- Thank you for your suggestion. Table 2 (Table 3 of the revised manuscript) has been modified comprehensively with additional information regarding various parameters including drug IDs, overall ADMET profile, Docking matrices, synthetic accessibility etc. Moreover, we provided a transparent justification for selecting the ZINC4004291 as the lead followed by multiple parameters including moderate docking score but stable pose, lowest predicted toxicity, favourable ADMET profile, compact MD behaviour, acceptable synthetic accessibility and commercial availability in ZINC database. Therefore, the lead selection was based on a multi-parameter decision matrix rather than docking score alone.

Reviewer comment 5: MD simulations and interpretation-

- Include time-resolved analyses (plots of RMSD, RMSF, radius of gyration, SASA, and interfacial hydrogen bonds) with statistics (means, standard deviations) over equilibrated windows.
- If feasible, report results from at least two independent simulations to assess reproducibility.
- Temper claims about binding stability and functional implications; discuss uncertainty and alternative interpretations, and clearly connect MD observations to potential VacA functional effects in a cautious, hypothesis-driven manner.
- Thank you for your valuable advice. According to the reviewer’s comment, the molecular dynamics (MD) analysis section has been revised to improve clarity and interpretational rigor. Specifically, the structural stability metrics of RMSDs are now reported as mean ± standard deviation calculated over the equilibrated simulation window (last 20 ns) to provide a more statistically robust interpretation which are included into the revised table (Table 3). Additionally, statements implying definitive conclusions about stability have been moderated; for example, phrases such as “confirmed stability” have been replaced with more cautious wording such as “suggested stable behaviour within simulation constraints” throughout the revised manuscript. Functional interpretations derived from the MD results are now explicitly framed as hypothesis-generating observations rather than definitive mechanistic conclusions. Finally, a dedicated paragraph discussing limitations of the MD simulations, including simulation timescale constraints and the absence of replicate trajectories, has been added to provide balanced interpretation and transparency.

Reviewer comment 6: ADMET, developability, and limitations

- Report exact ADMET predictions for all top ligands and the reference compound, with thresholds used for classification and a critical discussion of model limitations.
- Frame ZINC4004291 as a promising computational lead with the caveat that experimental evaluation is essential.
- Include a balanced developability discussion comparing top ligands on key properties and acknowledging limitations of in silico predictions.
- Taking your advice into careful consideration, exact ADMET values are now tabulated for all eight ligands as well as the reference compound, ranitidine. We also reframed ZINC4004291 as a promising computational lead and thus emphasized that it is a promising computational lead requiring experimental validation throughout the revised manuscript. Moreover, we clearly explicit the limitation of the constraints associated computational pharmacokinetic assessments into the limitation section.

 Reviewer comment 7: Biological relevance and context

- Strengthen the Discussion to articulate how occupancy of the modelled pocket could influence VacA function at the cellular level, including hypotheses about interference with receptor engagement, oligomerization, or pore formation. Tie these hypotheses to the most recent cryo-EM or structural work and provide precise citations.
- Thank you for your insightful observation. Now, the discussion has been expanded to better contextualize the biological relevance of the identified binding pocket in relation to VacA function. Specifically, the revised discussion section now integrates current knowledge on VacA receptor interactions, oligomerization dynamics, and pore formation mechanisms to provide a clearer functional framework for interpreting ligand binding within the predicted pocket. Based on these insights, potential disruption models are proposed to explain how ligand occupancy could interfere with VacA activity. These mechanistic interpretations are explicitly presented as hypotheses rather than definitive conclusions, and recent structural studies have been incorporated to support and contextualize the discussion.

 Reviewer comment 8: Nomenclature, figures, and supplementary materials

- Ensure consistent VacA domain nomenclature throughout (p55 vs. p55 domain) and accurate cross-referencing of figures and tables.
- Improve figure legends to be self-contained and consider adding a high-level schematic illustrating how ligand binding might affect VacA function, for readers accustomed to broader VacA biology.
- We extend our gratitude to you for providing valuable suggestions. The manuscript has been revised to improve clarity and consistency in nomenclature, figures, and supplementary materials. The terminology **“VacA p55 domain”** is now used consistently throughout the text. All figure references have been carefully checked and corrected where necessary, and figure legends have been rewritten to be fully self-contained and informative. In addition, we already have drawn the hypothesis associated with mechanistic implication of ligand-mediated VacA inhibition process in introduction section, although this knowledge is prediction driven as well as literature based. Thus, the overall mechanistic way of functioning of VacA p55 domain is yet to be understand and need more study to find the actual scenario which may be included in future.

**Minor revisions**

- Limitations and validation: Add a dedicated limitations subsection detailing docking caveats, MD interpretation caveats, and the need for experimental validation.
- Comparative context: If relevant VacA inhibitor literature exists, provide a brief positioning within the field and identify translational gaps or opportunities.
- We appreciate your valuable suggestion and thankful to you for giving us opportunity to improve our manuscript. In the revised manuscript, a dedicated subsection titled “Study Limitations” has been added dealing to discuss regarding docking scoring limitations, rigid receptor approximation, lack of replicate MD, no experimental validation (Line 753-766). Moreover, a brief literature-context paragraph has been incorporated to better position the present study within existing VacA research. The revised text compares our findings with previous computational investigations targeting VacA, highlighting similarities and methodological differences. It also identifies key translational gaps between computational predictions and experimental validation in current literature. Accordingly, the present work is positioned as a **hypothesis-generating study** that provides structural and mechanistic insights while emphasizing the need for subsequent experimental confirmation (Line 121-135).

**Reviewer 2:**

Comments to the Author

The study addresses an important problem—anti-virulence targeting of VacA as an alternative to antibiotics, and applies a standard CADD pipeline (homology modelling → docking → ADMET → MD simulation → PCA/FEL). The workflow is coherent and technically competent. However, several issues need strengthening, especially methodological justification and reproducibility.

- Thank you for complement and being positive to us.

Reviewer comment 1: Novelty and scope: Targeting VacA p55 domain via in silico screening has been reported previously. Then, what’s the novelty of this work?

- We appreciate this important concern. While previous studies have explored VacA as a potential drug target, our study differs in several aspects:

1. We performed **strain-wide genome annotation using RAST** to confirm conservation of VacA across multiple clinically relevant strains.
2. We applied a **systematic filtering pipeline**: 3000 ZINC15 compounds → active site binding → Lipinski filtering → ADMET profiling → MD simulation → PCA → FEL → MM/GBSA.
3. Unlike prior reports focusing mainly on docking, we incorporated 100 ns MD simulation, Principal Component Analysis (PCA), Free Energy Landscape (FEL) analysis, Mechanics Generalized Born Surface Area (MM/GBSA) along with Comparative ADMET profiling with a reference compound.

We have clarified this novelty in the Introduction and Discussion sections of our revised manuscript with proper citations.

Reviewer comment 2: Control molecule choice Ranitidine is used as a reference compound, but it is not a VacA inhibitor and does not mechanistically target VacA. Then why did you choose ranitidine as a control molecule?

- We do fully agree that Ranitidine is not a direct VacA inhibitor. It was selected as a **pharmacological reference compound** commonly used in gastric disorders to provide a comparative benchmark for docking and ADMET profiling. However, to avoid mechanistic misinterpretation, we have clearly stated that ranitidine **does not directly target VacA** and also clarified that it was used only for **comparative computational benchmarking.**

This clarification has also been added in the revised Methods (Line 201-203) and Limitation (Line 766-770) sections.

Reviewer comment 3: Docking protocols and reproducibility: Some essential details need to mention clearly. For example,

- Grid box center coordinates and dimensions
- Exhaustiveness value used in AutoDock Vina
- Whether protein flexibility was considered
- Docking validation strategy (re-docking, RMSD threshold)
- We thank the reviewer for highlighting this issue. The following details have now been added to the revised manuscript (Line 216-221):
- **Grid box center coordinates:** (5.63Å, 29.26Å and 22.13Å of X, Y and Z values respectively specified in revised Methods)
- **Grid box dimensions:** (size in 43.73Å, 65.95Å and 70.66 Å of X, Y and Z respectively)
- **Exhaustiveness value:** 8 (default AutoDock Vina parameter)
- **Energy range:** 3 Kcal/Mole
- **Number of modes:** 9
- **Protein flexibility:** Docking was performed using a rigid receptor model.
- **Docking validation:** Redocking of native ligand into the active site was performed, and RMSD < 2.0 Å confirmed docking reliability.

These additions improve reproducibility and transparency.

Reviewer comment 4: Binding affinity interpretation-

The docking score of ligand 8 is –6.9 kcal/mol, which is moderate, and not so strong. Several other ligands show higher affinity (e.g., –8.2, –7.8 kcal/mol) than ligand 8. Then why did you choose ligand 8 as potential therapeutics?

- Thank you for noticing an important observation. Although some ligands showed higher docking scores (–8.2, –7.8 kcal/mol), ligand 8 (-6.9 kcal/mol) was selected on the basis of several key factors including:

1. It exhibited the **most favourable ADMET profile**
2. It showed:
   - No AMES toxicity
   - No hERG inhibition
   - No hepatotoxicity
   - No skin sensitization
   - Other ligands with higher docking scores showed some sort of toxicity.
3. Demonstrated **stable MD behaviour**
4. Showed compactness and favourable conformational stability in PCA and FEL analyses

Thus, selection was based on a **multi-parameter decision**, not docking score alone. This justification has been expanded in the Discussion section.

Reviewer comment 5: MD simulation rigor-

Although MD simulation was performed rigorously with various parameters (e.g. 100 ns MD, RMSD, RMSF, Rg, SASA, H-bonds, PCA, FEL), there remains some concerns such as no replicate simulations were performed and no MM/PBSA or MM/GBSA binding free energy was analyzed.

- We appreciate your complements. we have now **performed MM/GBSA binding free energy calculations** for the VacA–lead complexes to provide an additional energetic evaluation of ligand binding stability. The **MM/GBSA analysis has been incorporated into the revised manuscript accordingly (Line 282-295 (Methods), 559-618 (Results), 723-728 (Discussion))**, and the calculated binding free energies are now reported and discussed to support the docking and molecular dynamics findings. These results provide further quantitative insight into the energetic favourability of the ligand–protein interactions enabling credential mechanistic approach.

Regarding replicate simulations, we acknowledged that **multiple independent MD replicates were not performed due to computational resource limitations (Line 756-757)**. This limitation has now been clearly stated in the **Limitation section**, and future work will incorporate replicate MD simulations to further strengthen the robustness of the dynamic analysis.

Reviewer comment 6: Overstatement of biological implications-

In the introduction, the statement implying “proposed inhibitors could prevent gastritis and peptic ulcers by preventing the *H. pylori* attachment, colonization, and survival in the host cells” is not experimentally validated and seems overstated. Replace “could prevent gastritis and peptic ulcers by preventing the *H. pylori* attachment, colonization, and survival in the host cells” with “may interfere with VacA-mediated host interactions and the survival of H. pylori, although the experimental validation remains pending.”

- We completely agree. The suggested wording has been incorporated verbatim in to the revised manuscript.

Reviewer comment 7: Title: “Structure-guided discovery” is suitable, but slightly overstated. Replace the title with “Structure-guided identification of potential inhibitors targeting the VacA toxin of Helicobacter pylori”.

- We are grateful for your suggestion. The title and short title have been revised accordingly (Line 1-3).

Reviewer comment 8: Other Suggestions:

- In Abstract, replace “has implicated the favorable level” with “indicated favorable levels”.
- Replace “optimum thermodynamic behaviour” with “favorable thermodynamic stability”.
- Use “molecular dynamics simulation” consistently instead of “molecular dynamic simulation”.
- Replace “Newzealand” with “New Zealand” Replace “Nizeria” with “Nigeria”.\
- All suggested corrections have been implemented:
- “has implicated the favorable level” → **“indicated favorable levels”**
- “optimum thermodynamic behaviour” → **“favorable thermodynamic stability”**
- Consistent use of **“molecular dynamics simulation”**
- “Newzealand” → **New Zealand**
- “Nizeria” → **Nigeria**

The manuscript has also undergone additional proofreading to improve clarity and consistency.

**Reviewer #3:** There are minor issues which the authors should address:

- We sincerely thank the reviewer for the careful evaluation of our manuscript and for the constructive suggestions. The comments have helped us improve the clarity, methodological rigor, and presentation of the study. All suggestions have been addressed as detailed below.

Reviewer comment 1: The "Introduction" section requires slight revisions to improve the literature review by offering a more precise and informative discussion. It should outline the strengths and limitations of existing approaches and clearly differentiate the proposed method. Additionally, the study’s motivation and contributions should be more explicitly stated.

- Thank you for this helpful suggestion. The **Introduction section has been revised and expanded** to provide a more structured and informative literature review. The updated text now discusses the strengths and limitations of previously reported computational and experimental approaches targeting H. pylori virulence factors, including VacA. In addition, the **motivation of the present study and its specific contributions namely the identification of potential inhibitors targeting the VacA p55 domain through integrated docking, molecular dynamics simulations, ADMET evaluation and free binding energy are now clearly as well as comprehensively stated.** These revisions improve the positioning of our work within the existing literature and clarify the novelty of the proposed computational strategy.

Reviewer comment 2: Result section: Please condense the Results section to focus solely on the outcomes of the study. Remove all literature-based statements from this section, as they should instead be addressed in the Discussion, where they can be linked to and interpreted in the context of the study’s findings.

- We thank the reviewer for this important suggestion which will help to further improve the quality of the revised manuscript. In the result section, we have tried to focus solely on the outcomes of the study and removed literature-based statements from this section. Moreover, we tried to link up these literature-based statements with the respected context of results in the discussion section.

Reviewer comment 3: Figures: The figures appear ordinary and lack adequate labeling. For Figure 2, please include proper labeling of the Ramachandran regions. For Figure 4, depict the active site residues within the figure rather than only mentioning the region in the caption. For Figure 8, visualization would be improved by presenting a 3D plot of the principal component analysis. Additionally, including 3D representations of the Gibbs free energy landscape would enhance clarity and interpretation.”

- We thank the reviewer for the valuable suggestions regarding figure quality and visualization.

Figure 2 (Ramachandran plot): The plot has been revised to include clear labeling of the favored, allowed, and disallowed regions, improving interpretability.

Figure 4 (Active site visualization): The figure has been updated to explicitly display and label the active site residues within the structural representation, rather than only describing them in the caption. Moreover, appropriate tunnel information also added to the updated figure and to the newly added table.

Figure 8 (PCA analysis): We appreciate the suggestion to include a 3D PCA representation. However, we have retained the 2D PCA projection (PC1 vs PC2), as it captures the majority of the essential conformational variance and allows clearer visualization of dominant motion clusters. In our case, the inclusion of a third principal component contributed minimally to the overall variance and introduced additional visual complexity without substantially improving interpretability. Therefore, to maintain clarity and consistency with common practices in molecular dynamics analysis, a 2D representation was considered more appropriate. We would like to address the similar observation regarding FEL plots as they are not in 3D representation. It was retrained as 2D plot as FEL lacked of PC3, thus 3D plots were not compatible with 2D PCA representation.

These revisions collectively enhance the clarity, labeling, and interpretability of the figures in the revised manuscript.

Reviewer comment 4: Recent advancements have positioned vaccine design as a leading technology for the prevention of infectious diseases, focusing on prophylactic measures rather than therapeutic intervention. In this context, it is important to justify the choice of a drug design approach over vaccine development. Although several studies have explored vaccine design targeting this bacterium, a rational discussion should be provided on the selection of the protein for drug design instead, citing relevant literature (<https://doi.org/10.1177/11779322251331104>). Additionally, the advantages of the proposed drug approach compared to existing vaccine strategies should be clearly highlighted.”

- We thank the reviewer for this important suggestion. A **new paragraph has been added to the Introduction and Discussion sections** addressing the rationale for pursuing a **drug design strategy targeting VacA** rather than a vaccine-based approach. While vaccine development represents a promising prophylactic strategy against H. pylori, several challenges remain, including antigenic variability, limited long-term protective efficacy, and difficulties in generating robust mucosal immune responses. In contrast, **targeting VacA with small-molecule inhibitors offers a complementary therapeutic approach capable of directly neutralizing toxin-mediated pathogenic effects during infection.** The revised manuscript now includes a discussion comparing these strategies to support the context of drug designing instead of vaccine (Line 731-742).

Reviewer comment 5: Please describe the hydrogen bonding interactions within the complexes over the course of the different simulation trajectories. Refer to the cited paper for guidance and ensure proper acknowledgment is provided (<https://journals.plos.org/plosone/article?id=10.1371/journal.pone.0326219>).

- We appreciate this insightful recommendation. In the revised manuscript, the **hydrogen bonding interactions between VacA and the selected ligands have now been analyzed throughout the molecular dynamics trajectories.** The results describing hydrogen bond persistence, occupancy, and temporal stability during the simulation have been added to the **MD simulation analysis subsection of the Results and Discussion sections (Line 555-564 (Results), 710-714 (Discussion)).** These findings help clarify the dynamic stability of ligand–protein interactions.

Reviewer comment 6: Please provide the DCCM analysis, Refer to the cited paper for guidance and ensure proper acknowledgment is provided (<https://journals.plos.org/plosone/article?id=10.1371/journal.pone.0339474>).

- We do appreciate reviewer for this insightful observation. While DCCM can provide additional insight into correlated residue motions, our study already includes PCA, FEL, and MM/GBSA analyses, which collectively capture the essential conformational dynamics, stability, and binding energetics of the system.

Given that PCA inherently reflects correlated atomic motions and FEL represents the underlying conformational landscape, we believe that these analyses sufficiently address the dynamic behaviours of the system. Therefore, DCCM analysis was not included to avoid redundancy. However, we agree that it may offer complementary information and will consider its inclusion in future work.
