# Supplementary material for: Structure-guided identification of a potential inhibitor targeting the VacA toxin of Helicobacter pylori
Source: PLoS One. 2026 Jul 22;21(7):e0354383. doi: 10.1371/journal.pone.0354383 (PMC13390867; doi:10.1371/journal.pone.0354383)
Supplement: Peer review history

# Author comment: Author response to Decision Letter 0

**DOI:** 10.1371/journal.pone.0354383.r001

**Transfer Alert**

This paper was transferred from another journal. As a result, its full editorial history (including decision letters, peer reviews and author responses) may not be present.

19 Aug 2025

# Peer review: Decision Letter 0

**DOI:** 10.1371/journal.pone.0354383.r002

**Authors:** Ahmed Elkamhawy

10 Feb 2026

PONE-D-25-45307

Structure-guided discovery of a novel inhibitor targeting VacA toxin of Heliobacter pylori

PLOS One

Dear Dr. Hossain,

Thank you for submitting your manuscript to PLOS ONE. After careful consideration, we feel that it has merit but does not fully meet PLOS ONE’s publication criteria as it currently stands. Therefore, we invite you to submit a revised version of the manuscript that addresses the points raised during the review process.

Please submit your revised manuscript by Mar 27 2026 11:59PM. If you will need more time than this to complete your revisions, please reply to this message or contact the journal office at plosone@plos.org. When you're ready to submit your revision, log on to https://www.editorialmanager.com/pone/ and select the 'Submissions Needing Revision' folder to locate your manuscript file.

Please include the following items when submitting your revised manuscript:

- A letter that responds to each point raised by the academic editor and reviewer(s). You should upload this letter as a separate file labeled 'Response to Reviewers'.
- A marked-up copy of your manuscript that highlights changes made to the original version. You should upload this as a separate file labeled 'Revised Manuscript with Track Changes'.
- An unmarked version of your revised paper without tracked changes. You should upload this as a separate file labeled 'Manuscript'.

If you would like to make changes to your financial disclosure, please include your updated statement in your cover letter. Guidelines for resubmitting your figure files are available below the reviewer comments at the end of this letter.

If applicable, we recommend that you deposit your laboratory protocols in protocols.io to enhance the reproducibility of your results. Protocols.io assigns your protocol its own identifier (DOI) so that it can be cited independently in the future. For instructions see: https://journals.plos.org/plosone/s/submission-guidelines#loc-laboratory-protocols. Additionally, PLOS ONE offers an option for publishing peer-reviewed Lab Protocol articles, which describe protocols hosted on protocols.io. Read more information on sharing protocols at https://plos.org/protocols?utm_medium=editorial-email&utm_source=authorletters&utm_campaign=protocols.

We look forward to receiving your revised manuscript.

Kind regards,

Ahmed Elkamhawy

Academic Editor

PLOS One

Journal Requirements:

When submitting your revision, we need you to address these additional requirements.

1. Please ensure that your manuscript meets PLOS ONE's style requirements, including those for file naming. The PLOS ONE style templates can be found at

https://journals.plos.org/plosone/s/file?id=wjVg/PLOSOne_formatting_sample_main_body.pdf and

https://journals.plos.org/plosone/s/file?id=ba62/PLOSOne_formatting_sample_title_authors_affiliations.pdf

2. Please note that PLOS One has specific guidelines on code sharing for submissions in which author-generated code underpins the findings in the manuscript. In these cases, we expect all author-generated code to be made available without restrictions upon publication of the work. Please review our guidelines at https://journals.plos.org/plosone/s/materials-and-software-sharing#loc-sharing-code and ensure that your code is shared in a way that follows best practice and facilitates reproducibility and reuse.

3. Thank you for stating the following in the Acknowledgments Section of your manuscript:

“This work was partially supported by Noakhali Science and Technology University ResearchCell Grant, 2022–23 (Grant No.: NSTU-RC-BGE-T-23-35).”.

We note that you have provided additional information within the Acknowledgements Section that is not currently declared in your Funding Statement. Please note that funding information should not appear in the Acknowledgments section or other areas of your manuscript. We will only publish funding information present in the Funding Statement section of the online submission form.

Please remove any funding-related text from the manuscript and let us know how you would like to update your Funding Statement. Currently, your Funding Statement reads as follows:

“The author(s) received no specific funding for this work.”

Please include your amended statements within your cover letter; we will change the online submission form on your behalf.

4. Thank you for stating the following financial disclosure:

“This work was partially supported by Noakhali Science and Technology University ResearchCell Grant, 2022–23 (Grant No.: NSTU-RC-BGE-T-23-35).”.

Please state what role the funders took in the study.  If the funders had no role, please state: "The funders had no role in study design, data collection and analysis, decision to publish, or preparation of the manuscript."

If this statement is not correct you must amend it as needed.

Please include this amended Role of Funder statement in your cover letter; we will change the online submission form on your behalf.

5. We note that the grant information you provided in the ‘Funding Information’ and ‘Financial Disclosure’ sections do not match.

When you resubmit, please ensure that you provide the correct grant numbers for the awards you received for your study in the ‘Funding Information’ section

6. Thank you for stating the following financial disclosure:

“The author(s) received no specific funding for this work.”

At this time, please address the following queries:

a)        Please clarify the sources of funding (financial or material support) for your study. List the grants or organizations that supported your study, including funding received from your institution.

b)        State what role the funders took in the study. If the funders had no role in your study, please state: “The funders had no role in study design, data collection and analysis, decision to publish, or preparation of the manuscript.”

c)        If any authors received a salary from any of your funders, please state which authors and which funders.

d)        If you did not receive any funding for this study, please state: “The authors received no specific funding for this work.”

Please include your amended statements within your cover letter; we will change the online submission form on your behalf.

7. If the reviewer comments include a recommendation to cite specific previously published works, please review and evaluate these publications to determine whether they are relevant and should be cited. There is no requirement to cite these works unless the editor has indicated otherwise.

[Note: HTML markup is below. Please do not edit.]

Reviewers' comments:

Reviewer's Responses to Questions

**Comments to the Author**

1. Is the manuscript technically sound, and do the data support the conclusions?

The manuscript must describe a technically sound piece of scientific research with data that supports the conclusions. Experiments must have been conducted rigorously, with appropriate controls, replication, and sample sizes. The conclusions must be drawn appropriately based on the data presented.

Reviewer #1: Partly

Reviewer #2: Yes

Reviewer #3: Yes

**********

2. Has the statistical analysis been performed appropriately and rigorously?

Reviewer #1: No

Reviewer #2: I Don't Know

Reviewer #3: Yes

**********

3. Have the authors made all data underlying the findings in their manuscript fully available?

The PLOS Data policy requires authors to make all data underlying the findings described in their manuscript fully available without restriction, with rare exception (please refer to the Data Availability Statement in the manuscript PDF file). The data should be provided as part of the manuscript or its supporting information, or deposited to a public repository. For example, in addition to summary statistics, the data points behind means, medians and variance measures should be available. If there are restrictions on publicly sharing data—e.g. participant privacy or use of data from a third party—those must be specified.

Reviewer #1: Yes

Reviewer #2: Yes

Reviewer #3: Yes

**********

4. Is the manuscript presented in an intelligible fashion and written in standard English?

PLOS ONE does not copyedit accepted manuscripts, so the language in submitted articles must be clear, correct, and unambiguous. Any typographical or grammatical errors should be corrected at revision, so please note any specific errors here.

Reviewer #1: Yes

Reviewer #2: Yes

Reviewer #3: Yes

**********

5. Review Comments to the Author

Please use the space provided to explain your answers to the questions above. You may also include additional comments for the author, including concerns about dual publication, research ethics, or publication ethics. (Please upload your review as an attachment if it exceeds 20,000 characters)

Reviewer #1: This work is timely and methodologically ambitious, presenting a coherent computational workflow that targets a virulence factor (VacA p55 domain) with potential translational relevance. However, in its current form the manuscript overstates translational readiness, provides limited methodological transparency, and lacks a sufficiently rigorous lead selection justification and experimental validation plan. A major revision is required to clarify scope, strengthen mechanistic framing, and ensure reproducibility before reconsideration for publication.

Major revisions

1. Translation, scope, and claims

• Reframe ZINC4004291 as an in silico hit rather than a drug candidate. Remove language implying immediate translational potential or efficacy, and explicitly state that no biochemical, cellular, or in vivo data are presented. Provide a clear plan for experimental validation and emphasize the predictive nature of the findings throughout.

• Include a precise statement of the manuscript’s scope: computational lead discovery with an explicit disclaimer that findings are predictive and require empirical validation.

2. Target rationale and mechanism

• Strengthen the biological rationale for targeting the p55 pocket. Clarify whether the pocket overlaps known functional residues involved in VacA membrane interaction, oligomerization, or pore formation, or if it represents a novel allosteric site. If possible, map the pocket onto established VacA functional interfaces and discuss potential mechanistic implications of ligand binding, with precise citations to the latest structural nad functional VacA literature.

3. Methods transparency and reproducibility

Add a comprehensive methods section with enough detail to enable replication:

• Docking: specify software/version, scoring function, grid center and dimensions, exhaustiveness, number of poses retained per ligand, receptor/ligand preparation steps, and how flexibility was treated (rigid receptor vs. induced-fit considerations).

• MD: specify force field, water model, simulation box type and size, ion concentration, time steps, thermostat settings, equilibration length, production run length, convergence criteria; report whether multiple independent runs were performed.

• Data and scripts: provide supplementary files with docking parameters, input files, and analysis scripts; include a brief description of reproducibility checks (e.g., replicate runs, sensitivity analyses).

• Include a comprehensive table for the top ligands (at least the eight initially highlighted) with docking scores (mean and range), key interactions, and basic physicochemical properties. Explain decisions to deprioritize ligands with similar or better docking scores but unfavorable ADMET, stability, or synthetic feasibility.

4. Lead selection and comparative data

• Present a side-by-side comparison of all top candidates using objective criteria:

• Docking metrics (scores, pose stability)

• MD stability indicators (average RMSD/RMSF in the binding region, convergence behavior across replicas if performed)

• ADMET predictions with explicit thresholds and caveats

• Practical considerations (synthetic accessibility, commercial availability)

• Provide a transparent justification for selecting ZINC4004291 as the lead, ensuring the rationale is not solely based on a single docking score.

5. MD simulations and interpretation

• Include time-resolved analyses (plots of RMSD, RMSF, radius of gyration, SASA, and interfacial hydrogen bonds) with statistics (means, standard deviations) over equilibrated windows. If feasible, report results from at least two independent simulations to assess reproducibility.

• Temper claims about binding stability and functional implications; discuss uncertainty and alternative interpretations, and clearly connect MD observations to potential VacA functional effects in a cautious, hypothesis-driven manner.

6. ADMET, developability, and limitations

• Report exact ADMET predictions for all top ligands and the reference compound, with thresholds used for classification and a critical discussion of model limitations. Frame ZINC4004291 as a promising computational lead with the caveat that experimental evaluation is essential.

• Include a balanced developability discussion comparing top ligands on key properties and acknowledging limitations of in silico predictions.

7. Biological relevance and context

• Strengthen the Discussion to articulate how occupancy of the modeled pocket could influence VacA function at the cellular level, including hypotheses about interference with receptor engagement, oligomerization, or pore formation. Tie these hypotheses to the most recent cryo-EM or structural work and provide precise citations.

8. Nomenclature, figures, and supplementary materials

• Ensure consistent VacA domain nomenclature throughout (p55 vs. p55 domain) and accurate cross-referencing of figures and tables.

• Improve figure legends to be self-contained and consider adding a high-level schematic illustrating how ligand binding might affect VacA function, for readers accustomed to broader VacA biology.

Minor revisions

• Limitations and validation: Add a dedicated limitations subsection detailing docking caveats, MD interpretation caveats, and the need for experimental validation.

• Comparative context: If relevant VacA inhibitor literature exists, provide a brief positioning within the field and identify translational gaps or opportunities.

Reviewer #2: The study addresses an important problem—anti-virulence targeting of VacA as an alternative to antibiotics, and applies a standard CADD pipeline (homology modeling → docking → ADMET → MD simulation → PCA/FEL). The workflow is coherent and technically competent. However, several issues need strengthening, especially methodological justification and reproducibility.

1. Novelty and scope: Targeting VacA p55 domain via in silico screening has been reported previously. Then, what’s the novelty of this work?

2. Control molecule choice Ranitidine is used as a reference compound, but it is not a VacA inhibitor and does not mechanistically target VacA. Then why did you choose ranitidine as a control molecule?

3. Docking protocols and reproducibility

Some essential details need to mention clearly. For example,

• Grid box center coordinates and dimensions

• Exhaustiveness value used in AutoDock Vina

• Whether protein flexibility was considered

• Docking validation strategy (re-docking, RMSD threshold)

4. Binding affinity interpretation

The docking score of ligand 8 is –6.9 kcal/mol, which is moderate, and not so strong. Several other ligands show higher affinity (e.g., –8.2, –7.8 kcal/mol) than ligand 8. Then why did you choose ligand 8 as potential therapeutics?

5. MD simulation rigor

Although MD simulation was performed rigorously with various parameters (e.g. 100 ns MD, RMSD, RMSF, Rg, SASA, H-bonds, PCA, FEL), there remains some concerns such as no replicate simulations were performed and no MM/PBSA or MM/GBSA binding free energy was analyzed.

6. Overstatement of biological implications

In the introduction, the statement implying “proposed inhibitors could prevent gastritis and peptic ulcers by preventing the H. pylori attachment, colonization, and survival in the host cells” is not experimentally validated and seems overstated. Replace “could prevent gastritis and peptic ulcers by preventing the H. pylori attachment, colonization, and survival in the host cells” with “may interfere with VacA-mediated host interactions and the survival of H. pylori, although the experimental validation remains pending.”

7. Title: “Structure-guided discovery” is suitable, but slightly overstated. Replace the title with “Structure-guided identification of potential inhibitors targeting the VacA toxin of Helicobacter pylori”.

8. Other Suggestions: In Abstract, replace “has implicated the favorable level” with “indicated favorable levels”.

• Replace “optimum thermodynamic behavior” with “favorable thermodynamic stability”.

• Use “molecular dynamics simulation” consistently instead of “molecular dynamic simulation”.

• Replace “Newzealand” with “New Zealand”

• Replace “Nizeria” with “Nigeria”

Reviewer #3: there are minor issues which the authors should address:

1. The "Introduction" section requires slight revisions to improve the literature review by offering a more precise and informative discussion. It should outline the strengths and limitations of existing approaches and clearly differentiate the proposed method. Additionally, the study’s motivation and contributions should be more explicitly stated.

2. Result section: Please condense the Results section to focus solely on the outcomes of the study. Remove all literature-based statements from this section, as they should instead be addressed in the Discussion, where they can be linked to and interpreted in the context of the study’s findings.

3. Figures: The figures appear ordinary and lack adequate labeling. For Figure 2, please include proper labeling of the Ramachandran regions. For Figure 4, depict the active site residues within the figure rather than only mentioning the region in the caption. For Figure 8, visualization would be improved by presenting a 3D plot of the principal component analysis. Additionally, including 3D representations of the Gibbs free energy landscape would enhance clarity and interpretation.”

4. Recent advancements have positioned vaccine design as a leading technology for the prevention of infectious diseases, focusing on prophylactic measures rather than therapeutic intervention. In this context, it is important to justify the choice of a drug design approach over vaccine development. Although several studies have explored vaccine design targeting this bacterium, a rational discussion should be provided on the selection of the protein for drug design instead, citing relevant literature (https://doi.org/10.1177/11779322251331104). Additionally, the advantages of the proposed drug approach compared to existing vaccine strategies should be clearly highlighted.”

5. Please describe the hydrogen bonding interactions within the complexes over the course of the different simulation trajectories. Refer to the cited paper for guidance and ensure proper acknowledgment is provided (https://journals.plos.org/plosone/article?id=10.1371/journal.pone.0326219).

6. Please provide the DCCM analysis, Refer to the cited paper for guidance and ensure proper acknowledgment is provided (https://journals.plos.org/plosone/article?id=10.1371/journal.pone.0339474)

**********

6. PLOS authors have the option to publish the peer review history of their article (what does this mean?). If published, this will include your full peer review and any attached files.

If you choose “no”, your identity will remain anonymous but your review may still be made public.

**Do you want your identity to be public for this peer review?**  For information about this choice, including consent withdrawal, please see our Privacy Policy.

Reviewer #1: No

Reviewer #2: **Yes:**  Shipan Das Gupta

Reviewer #3: **Yes:**  AMAM Zonaed Siddiki

**********

[NOTE: If reviewer comments were submitted as an attachment file, they will be attached to this email and accessible via the submission site. Please log into your account, locate the manuscript record, and check for the action link "View Attachments". If this link does not appear, there are no attachment files.]

To ensure your figures meet our technical requirements, please review our figure guidelines: https://journals.plos.org/plosone/s/figures

You may also use PLOS’s free figure tool, NAAS, to help you prepare publication quality figures: https://journals.plos.org/plosone/s/figures#loc-tools-for-figure-preparation.

NAAS will assess whether your figures meet our technical requirements by comparing each figure against our figure specifications.

Attachment Submitted filename: DSDG-Review-PONE.docx

# Author comment: Author response to Decision Letter 1

**DOI:** 10.1371/journal.pone.0354383.r003

17 May 2026

Dear Editor

We are grateful for your efforts in editing our manuscript and excellent comments and suggestions for us to revise and resubmit our manuscript. We have read carefully reviewers’ all comments and tried our best to revise our manuscript considering all comments and suggestions. Details revision are given below.

Editorial Comments

1. Please ensure that your manuscript meets PLOS ONE's style requirements, including those for file naming. The PLOS ONE style templates can be found at https://journals.plos.org/plosone/s/file?id=wjVg/PLOSOne_formatting_sample_main_body.pdf andhttps://journals.plos.org/plosone/s/file?id=ba62/PLOSOne_formatting_sample_title_authors_affiliations.pdf

► We sincerely thank the editor for the suggestion. We went through the manuscript and have carefully revised it according to the requirements of PLOS ONE’s style.

2. Please note that PLOS One has specific guidelines on code sharing for submissions in which author-generated code underpins the findings in the manuscript. In these cases, we expect all author-generated code to be made available without restrictions upon publication of the work. Please review our guidelines at https://journals.plos.org/plosone/s/materials-and-software-sharing#loc-sharing-code and ensure that your code is shared in a way that follows best practice and facilitates reproducibility and reuse.

► We did not generate any code to get access to the information of our manuscript. All information and data of our manuscript are available in the manuscript and supplementary information.

3. Thank you for stating the following in the Acknowledgments Section of your manuscript: “This work was partially supported by Noakhali Science and Technology University ResearchCell Grant, 2022–23 (Grant No.: NSTU-RC-BGE-T-23-35).” We note that you have provided additional information within the Acknowledgements Section that is not currently declared in your Funding Statement. Please note that funding information should not appear in the Acknowledgments section or other areas of your manuscript. We will only publish funding information present in the Funding Statement section of the online submission form. Please remove any funding-related text from the manuscript and let us know how you would like to update your Funding Statement. Currently, your Funding Statement reads as follows: “The author(s) received no specific funding for this work.” Please include your amended statements within your cover letter; we will change the online submission form on your behalf.

► We are thankful to the editor for the suggestion. According to the suggestion, we have removed the funding related statement “This work was partially supported by Noakhali Science and Technology University Research Cell Grant, 2022–23 (Grant No.: NSTU-RC-BGE-T-23-35)” from the Acknowledgements Section in our revised manuscript. The Funding Statement will be “The author(s) received no specific funding for this work.” Actually, we didn’t receive any specific funding for this research. However, we used a little money from the said grant to repair the computer facility of Bioinformatics and Computational Biology Laboratory of the Department of Biotechnology and Genetic Engineering, Noakhali Science and Technology University”. Thus, we had mentioned this in the Acknowledgements Section of the manuscript and we have already removed this information from the revised manuscript according to the given suggestion.

4. Thank you for stating the following financial disclosure: “This work was partially supported by Noakhali Science and Technology University ResearchCell Grant, 2022–23 (Grant No.: NSTU-RC-BGE-T-23-35).” Please state what role the funders took in the study. If the funders had no role, please state: "The funders had no role in study design, data collection and analysis, decision to publish, or preparation of the manuscript." If this statement is not correct you must amend it as needed. Please include this amended Role of Funder statement in your cover letter; we will change the online submission form on your behalf.

► Thank you very much for the suggestion. "The funders had no role in study design, data collection and analysis, decision to publish, or preparation of the manuscript."

5. We note that the grant information you provided in the ‘Funding Information’ and ‘Financial Disclosure’ sections do not match. When you resubmit, please ensure that you provide the correct grant numbers for the awards you received for your study in the ‘Funding Information’ section.

► The authors would like to pay their sincere gratitude to the editor. Actually, we didn’t receive any specific funding for this work. Hence, we have removed the funding information in our revised manuscript.

6. Thank you for stating the following financial disclosure: “The author(s) received no specific funding for this work.” At this time, please address the following queries:

a) Please clarify the sources of funding (financial or material support) for your study. List the grants or organizations that supported your study, including funding received from your institution.

► This work was partially supported by Noakhali Science and Technology University Research Cell Grant, 2022–23 (Grant No.: NSTU-RC-BGE-T-23-35). There was no specific funding for this study. However, a small amount of money from the said grant was utilized to repair the computer facilities of the Bioinformatics and Computational Biology Laboratory (of the Department of Biotechnology and Genetic Engineering, Noakhali Science and Technology University) used for this study.

b) State what role the funders took in the study. If the funders had no role in your study, please state: “The funders had no role in study design, data collection and analysis, decision to publish, or preparation of the manuscript.”

► The funders had no role in study design, data collection and analysis, decision to publish, or preparation of the manuscript.

c) If any authors received a salary from any of your funders, please state which authors and which funders.

► No author received salary from funders for this study.

d) If you did not receive any funding for this study, please state: “The authors received no specific funding for this work.”

► The authors received no specific funding for this work.

Please include your amended statements within your cover letter; we will change the online submission form on your behalf.

► The authors would like to pay their sincere thanks to the editor for the recommendation and suggestion. We have mentioned the role of the funders and the funding statement in the cover letter as suggested

7. If the reviewer comments include a recommendation to cite specific previously published works, please review and evaluate these publications to determine whether they are relevant and should be cited. There is no requirement to cite these works unless the editor has indicated otherwise.

► Thank you very much for the suggestion. We addressed all the comments from the reviewers carefully. Thus, we have made changes in the revised manuscript and used a few new references. We have also carefully evaluated the suggested previously published works and cited wherever it is necessary.

Revision of manuscript

Comments to the Author:

Reviewer #1:

This work is timely and methodologically ambitious, presenting a coherent computational workflow that targets a virulence factor (VacA p55 domain) with potential translational relevance. However, in its current form the manuscript overstates translational readiness, provides limited methodological transparency, and lacks a sufficiently rigorous lead selection justification and experimental validation plan. A major revision is required to clarify scope, strengthen mechanistic framing, and ensure reproducibility before reconsideration for publication.

► We sincerely thank the reviewer for the thoughtful, detailed, and constructive critique. The comments have helped to significantly improved the scientific rigor, clarity, and scope of our manuscript. We have carefully revised the manuscript to address all concerns, including reframing translational claims, strengthening methodological transparency, clarifying mechanistic rationale, and expanding reproducibility details.

Major revisions

Reviewer comment 1: Reframe ZINC4004291 as an in silico hit rather than a drug candidate. Remove language implying immediate translational potential and clearly state that findings are predictive. Provide an experimental validation plan.

► We do fully agree. In the revised manuscript, ZINC4004291 is consistently described as computationally identified in silico hit. In the Abstract section of our revised manuscript, we mentioned “Thus, the ligand ZINC4004291 is a computationally identified in silico hit for a possible drug to treat H. pylori infection, while additional experimental validation required” (Page 2, Line 42-44). Moreover, all statements implying translational readiness or therapeutic efficacy have been removed or softened. Additionally, a new Scope Statement has been added at the end of the Introduction as this study presents a computational lead discovery framework targeting the VacA p55 domain. The findings are predictive in nature and require biochemical, cellular, and in vivo validation (Line 152-154). A few sentences describing the “Proposed Experimental Validation Strategy” has also been added at the end of the Discussion section as per your suggestion (Page 28, Line 742-751).

Reviewer comment 2: Target rationale and mechanism-

Strengthen the biological rationale for targeting the p55 pocket. Clarify whether the pocket overlaps known functional residues involved in VacA membrane interaction, oligomerization, or pore formation, or if it represents a novel allosteric site. If possible, map the pocket onto established VacA functional interfaces and discuss potential mechanistic implications of ligand binding, with precise citations to the latest structural and functional VacA literature.

► Thank you for your valuable suggestions. We have substantially strengthened this section.

The CASTp-identified pocket was mapped onto known p55 structural interfaces based on PDB: 2QV3 and recent structural literature. Moreover, additional allosteric sites along with pocket were newly investigated by PrankWeb tools (Table 2 and Line 356-363). This step clarified regarding known functional residues corresponding to their position. Hence, the revised Discussion now clarifies the following mechanistic implications of ligand binding (Line 632-641).

• The p55 domain mediates receptor interaction and oligomerization.

• The identified pocket overlaps residues implicated in membrane interaction and structural stabilization.

• The pocket may represent a surface-exposed functional interface rather than a catalytic site.

Hence, now we cautiously stated: “Ligand occupancy within this pocket may interfere with VacA structural assembly or host receptor engagement, although this hypothesis requires experimental validation” in the discussion section (Line 640-641). Furthermore, recent structural and cryo-EM studies have been cited to contextualize the mechanistic hypothesis.

Reviewer comment 3: Methods transparency and reproducibility-

Add a comprehensive methods section with enough detail to enable replication:

• Docking: specify software/version, scoring function, grid center and dimensions, exhaustiveness, number of poses retained per ligand, receptor/ligand preparation steps, and how flexibility was treated (rigid receptor vs. induced-fit considerations).

• MD: specify force field, water model, simulation box type and size, ion concentration, time steps, thermostat settings, equilibration length, production run length, convergence criteria; report whether multiple independent runs were performed.

• Data and scripts: provide supplementary files with docking parameters, input files, and analysis scripts; include a brief description of reproducibility checks (e.g., replicate runs, sensitivity analyses).

• Include a comprehensive table for the top ligands (at least the eight initially highlighted) with docking scores (mean and range), key interactions, and basic physicochemical properties. Explain decisions to deprioritize ligands with similar or better docking scores but unfavorable ADMET, stability, or synthetic feasibility.

► Thank you for your insightful observations. We have significantly expanded the method section with enough details ensuring reproducibility. We have added the following information to the revised manuscript:

Docking Details (Line 216-221):

• Software: AutoDock Vina (version specified)

• Grid center coordinates (X, Y, Z values provided)

• Grid dimensions (Å specified)

• Exhaustiveness: 8

• Number of poses retained per ligand: 9

• Receptor preparation: removal of water, addition of hydrogens, Gasteiger charges

• Ligand preparation: energy minimization via Open Babel

• Flexibility: rigid receptor docking

• Docking validation: redocking RMSD < 2.0 Å

MD Simulation Details:

• GROMACS version specified

• Force field: CHARMM27

• Water model: TIP3P

• Box type: cubic

• Box distance: 1 nm

• Ion concentration: 0.15 M NaCl

• Time step: 2 fs

• NVT equilibration: 100 ps

• NPT equilibration: 100 ps

• Production run: 100 ns

• Thermostat: V-rescale

• Barostat: Parrinello–Rahman (Line 252-253)

However, Single trajectory simulations were performed; replicate simulations were not conducted due to computational constraints. We stated this under the limitation section.

Moreover, Docking parameter files, Coordinate files, MD input files including .xtc, .cpt and .mdp along with input files of the MM/GBSA calculation are available upon request to ensure public access and reproducibility.

Furthermore, we have comprehensively modified Table 2 (Table 3 in our revised manuscript) for the top ligands with docking scores, key interactions, and basic physicochemical properties.

Reviewer comment 4: Lead selection and comparative data-

• Present a side-by-side comparison of all top candidates using objective criteria:

• Docking metrics (scores, pose stability)

• MD stability indicators (average RMSD/RMSF in the binding region, convergence behaviour across replicas if performed)

• ADMET predictions with explicit thresholds and caveats

• Practical considerations (synthetic accessibility, commercial availability)

• Provide a transparent justification for selecting ZINC4004291 as the lead, ensuring the rationale is not solely based on a single docking score.

► Thank you for your suggestion. Table 2 (Table 3 of the revised manuscript) has been modified comprehensively with additional information regarding various parameters including drug IDs, overall ADMET profile, Docking matrices, synthetic accessibility etc. Moreover, we provided a transparent justification for selecting the ZINC4004291 as the lead followed by multiple parameters including moderate docking score but stable pose, lowest predicted toxicity, favourable ADMET profile, compact MD behaviour, acceptable synthetic accessibility and commercial availability in ZINC database. Therefore, the lead selection was based on a multi-parameter decision matrix rather than docking score alone.

Reviewer comment 5: MD simulations and interpretation-

• Include time-resolved analyses (plots of RMSD, RMSF, radius of gyration, SASA, and interfacial hydrogen bonds) with statistics (means, standard deviations) over equilibrated windows.

• If feasible, report results from at least two independent simulations to assess reproducibility.

• Temper claims about binding stability and functional implications; discuss uncertainty and alternative interpretations, and clearly connect MD observations to potential VacA functional effects in a cautious, hypothesis-driven manner.

► Thank you for your valuable advice. According to the reviewer’s comment, the molecular dynamics (MD) analysis section has been revised to improve clarity and interpretational rigor. Specifically, the structural stability metrics of RMSDs are now reported as mean ± standard deviation calculated over the equilibrated simulation window (last 20 ns) to provide a more statistically robust interpretation which are included into the revised table (Tabl

Attachment Submitted filename: 17042026-Response_to_Reviewers.docx

# Peer review: Decision Letter 1

**DOI:** 10.1371/journal.pone.0354383.r004

**Authors:** Wagdy Eldehna

8 Jul 2026

<p>Structure-guided identification of a potential inhibitor targeting the VacA toxin of Helicobacter pylori

PONE-D-25-45307R1

Dear Dr. Hossain,

We’re pleased to inform you that your manuscript has been judged scientifically suitable for publication and will be formally accepted for publication once it meets all outstanding technical requirements.

Within one week, you’ll receive an e-mail detailing the required amendments. When these have been addressed, you’ll receive a formal acceptance letter and your manuscript will be scheduled for publication.

An invoice will be generated when your article is formally accepted. Please note, if your institution has a publishing partnership with PLOS and your article meets the relevant criteria, all or part of your publication costs will be covered. Please make sure your user information is up-to-date by logging into Editorial Manager at Editorial Manager® and clicking the ‘Update My Information' link at the top of the page. For questions related to billing, please contact billing support.

If your institution or institutions have a press office, please notify them about your upcoming paper to help maximize its impact. If they’ll be preparing press materials, please inform our press team as soon as possible -- no later than 48 hours after receiving the formal acceptance. Your manuscript will remain under strict press embargo until 2 pm Eastern Time on the date of publication. For more information, please contact onepress@plos.org.

Kind regards,

Wagdy M. Eldehna, Ph.d

Academic Editor

PLOS One

Additional Editor Comments (optional):

Reviewers' comments:

Reviewer's Responses to Questions

**Comments to the Author**

1. If the authors have adequately addressed your comments raised in a previous round of review and you feel that this manuscript is now acceptable for publication, you may indicate that here to bypass the “Comments to the Author” section, enter your conflict of interest statement in the “Confidential to Editor” section, and submit your "Accept" recommendation.

Reviewer #2: All comments have been addressed

Reviewer #3: All comments have been addressed

**********

2. Is the manuscript technically sound, and do the data support the conclusions?

The manuscript must describe a technically sound piece of scientific research with data that supports the conclusions. Experiments must have been conducted rigorously, with appropriate controls, replication, and sample sizes. The conclusions must be drawn appropriately based on the data presented.

Reviewer #2: Yes

Reviewer #3: Yes

**********

3. Has the statistical analysis been performed appropriately and rigorously?

Reviewer #2: N/A

Reviewer #3: Yes

**********

4. Have the authors made all data underlying the findings in their manuscript fully available?

The PLOS Data policy requires authors to make all data underlying the findings described in their manuscript fully available without restriction, with rare exception (please refer to the Data Availability Statement in the manuscript PDF file). The data should be provided as part of the manuscript or its supporting information, or deposited to a public repository. For example, in addition to summary statistics, the data points behind means, medians and variance measures should be available. If there are restrictions on publicly sharing data—e.g. participant privacy or use of data from a third party—those must be specified.

Reviewer #2: Yes

Reviewer #3: Yes

**********

5. Is the manuscript presented in an intelligible fashion and written in standard English?

PLOS ONE does not copyedit accepted manuscripts, so the language in submitted articles must be clear, correct, and unambiguous. Any typographical or grammatical errors should be corrected at revision, so please note any specific errors here.

Reviewer #2: Yes

Reviewer #3: Yes

**********

6. Review Comments to the Author

Please use the space provided to explain your answers to the questions above. You may also include additional comments for the author, including concerns about dual publication, research ethics, or publication ethics. (Please upload your review as an attachment if it exceeds 20,000 characters)

Reviewer #2: The authors have adequately addressed all of the comments and recommendations provided by me and the other reviewers. The quality of the manuscript has improved significantly following these revisions. Therefore, I have no further comments and recommend that the manuscript be accepted for publication.

Reviewer #3: The article can be accepted in its current form. all queries have been met by the authors. .............

**********

7. PLOS authors have the option to publish the peer review history of their article (what does this mean?). If published, this will include your full peer review and any attached files.

If you choose “no”, your identity will remain anonymous but your review may still be made public.

**Do you want your identity to be public for this peer review?**  For information about this choice, including consent withdrawal, please see our Privacy Policy.

Reviewer #2: **Yes:**  Shipan Das Gupta

Reviewer #3: **Yes:**  AMAM Zonaed Siddiki

**********

# Editor report: Acceptance letter

**DOI:** 10.1371/journal.pone.0354383.r005

**Authors:** Wagdy Eldehna

PONE-D-25-45307R1

PLOS One

Dear Dr. Hossain,

I'm pleased to inform you that your manuscript has been deemed suitable for publication in PLOS One. Congratulations! Your manuscript is now being handed over to our production team.

At this stage, our production department will prepare your paper for publication. This includes ensuring the following:

* All references, tables, and figures are properly cited

* All relevant supporting information is included in the manuscript submission,

* There are no issues that prevent the paper from being properly typeset

You will receive further instructions from the production team, including instructions on how to review your proof when it is ready. Please keep in mind that we are working through a large volume of accepted articles, so please give us a few days to review your paper and let you know the next and final steps.

Lastly, if your institution or institutions have a press office, please let them know about your upcoming paper now to help maximize its impact. If they'll be preparing press materials, please inform our press team within the next 48 hours. Your manuscript will remain under strict press embargo until 2 pm Eastern Time on the date of publication. For more information, please contact onepress@plos.org.

You will receive an invoice from PLOS for your publication fee after your manuscript has reached the completed accept phase. If you receive an email requesting payment before acceptance or for any other service, this may be a phishing scheme. Learn how to identify phishing emails and protect your accounts at https://explore.plos.org/phishing.

If we can help with anything else, please email us at customercare@plos.org.

Thank you for submitting your work to PLOS ONE and supporting open access.

Kind regards,

PLOS ONE Editorial Office Staff

on behalf of

Dr. Wagdy M. Eldehna

Academic Editor

PLOS One
